# Basic primitives for molecular diagram sketching

**DOI:** 10.1186/1758-2946-2-8

**Published:** 2010-10-05

**Authors:** Alex M Clark

**Affiliations:** 1 Molecular Materials Informatics, Inc, Montréal, QC, Canada

## Abstract

A collection of primitive operations for molecular diagram sketching has been developed. These primitives compose a concise set of operations which can be used to construct publication-quality 2 D coordinates for molecular structures using a bare minimum of input bandwidth. The input requirements for each primitive consist of a small number of discrete choices, which means that these primitives can be used to form the basis of a user interface which does not require an accurate pointing device. This is particularly relevant to software designed for contemporary mobile platforms. The reduction of input bandwidth is accomplished by using algorithmic methods for anticipating probable geometries during the sketching process, and by intelligent use of template grafting. The algorithms and their uses are described in detail.

## Introduction

Molecular structure diagrams have been the mainstay of chemical communication since molecules began to be rationalised as Lewis structures. The basic ideas involved in representing structures have proven to be remarkably resilient [1-3]. While typographic conventions used in the late 19th century differ slightly from modern publications, organic structures are mostly quite recognisable when compared to literature published more than a century ago.

In recent decades, the process of creating molecular structure diagrams has steadily shifted towards use of computer software, which is now used exclusively for publications, and is in the process of replacing hand-drawings by way of electronic lab notebooks [4]. There is now an abundance of software packages which allow the user to sketch a molecular diagram on a desktop or laptop computer, using the mouse and keyboard to specify the content and geometry of the molecule [5,6]. There are also a number of algorithms in general use which allow molecular structure diagrams to be produced automatically using only the molecular topology [7-10]. The availability of information relating molecular connection tables to important properties has made possible the rise of the subfield of computational chemistry commonly known as cheminformatics [11,12], and the usual data entry method for chemical structures is via software designed for sketching 2 D diagrams of molecules.

The subject of this work is a slightly different perspective on the drawing of a 2 D molecular structure diagram. Assuming that the composition of the molecule is known, and the desired output is an arrangement of atoms and bonds onto a flat surface, the process of building up the diagram can be described as a series of primitive unit steps, leading to a molecular connection table, with 2 D coordinates for each atom [13].

In this work, we will explore an alphabet of primitives which has been chosen for the following properties:

- small number of operation types

- minimal degrees of freedom for input

- opportunities for automated inference

The primary motivation for abstracting the sketching process in such a way is the emergence of new hardware devices which are highly constrained in terms of user input, such as smartphones, tablets and netbooks. These devices often lack an accurate pointing device. Mobile devices with touch screens, for example, are effective for selecting objects, but they are much less effective for the precise positioning operations upon which conventional molecule drawing software relies. Mobile devices which lack a touch screen offer merely a keypad and directional cursor keys.

By describing all of the unit primitives needed to produce a molecular structure diagram in such a way that none of them requires precise user-supplied position information, structure sketching becomes possible for environments in which the user input is limited to little more than a series of menu choices.

## Experimental

The objective of this work is to describe a collection of primitive drawing operations which provides a comprehensive set of editing capabilities. These can be used to compose complex diagrams with minimal effort on behalf of the operator.

Ideally, each primitive would be able to examine the molecule diagram thus far, determine what it is the user wants changed, and execute the change. In practice, several additional fields are required for most operations. The unit primitives which are described in this work operate as sequences of:

- select **subject**

- select **action**

- select **result**

The **subject **is an annotation to the existing structure, which consists of:

- current atom or current bond

- a set of selected atoms

At any time there may be a current atom or a current bond, but not both. Each atom of the existing structure is either selected or unselected. In the text that follows, the term *subject atoms *is defined as a set of:

- all selected atoms, if there are any;

- *or: *the current atom, if there is one;

- *or*: the two atoms of the current bond, if there is one;

- *else*: an empty set.

The **action **is the choice of primitive to apply to the current subject.

For many primitives, there is just one possible outcome when applied to a particular subject, e.g. changing atoms to a specific element, for which it is appropriate to design the primitive to have an unambiguous effect. For some of the more complicated primitives, there could be more than one possible outcome, e.g. attaching a template with multiple possible geometries. In these cases, the primitive may choose to generate a number of **results**. The list of results should be sorted so that the most plausible result is first, and the remaining possibilities in order of decreasing relevance.

When this scheme is mapped to a practical implementation of a user interface, the sequence can be described as:

- select the atoms or bonds of interest

- pick the action from a menu

- if there is more than one result, and the best suggestion is not the desired one, pick from the list of possibilities

The remainder of this section describes a minimal set of primitive classes which provide enough functionality to assemble a fully-featured molecular drawing package.

## Atoms

Atom modification primitives are mostly straightforward and unambiguous, such as changing an element label, or altering atom-centred properties such as charge or radical count. The number of primitives required depends on the number of editable atom properties used to describe the molecular structure. The following primitive classes are representative.

### Add Atom

A new atom is created. Its position is determined automatically. If the structure already contains one or more atoms, it is along the top and to the right of the existing atoms.

One primitive is required for each element of the periodic table. The new atom will have the corresponding label, with default values for all other properties, and no bonds.

### Set Element Label

The subject atoms have their atomic symbol changed. One primitive is required for each element of the periodic table. If there is no subject, then this primitive should be mapped to the corresponding *Add Atom *primitive.

Variations on this primitive should allow arbitrary values to be entered via an input dialog, for symbols which are not atomic elements, such as abbreviations or Markush structures.

### Set Ionic Charge

The subject atoms have their ionic charge set to a specific value, such as 0, -1, +1, etc. The primitives in this class can either specify exact values for the charge, or they can be increment/decrement operations.

### Set Unpaired Electrons

The subject atoms have the number of unpaired electrons (also known as *radical count*) set to a specific value, where 0 is for diamagnetic atoms, 1 is for radicals, 2 is for carbene-like species, etc. One primitive is needed for each available value.

### Set Hydrogen Count

Some molecular structure formats allow the number of implicit hydrogens to be specified [14]. The default value should be *automatic*, whereby the number of implied hydrogens is calculated from the atomic element, charge, unpaired electron count and immediate bonding environment. A value of 0 implies that no additional hydrogens are attached, and a value of greater than zero specifies exactly how many are present. One primitive is needed for each possible value.

### Set Isotope

The molecular mass of an atom defaults to the statistical average of its natural abundance. An atom can alternatively be defined to be a specific isotope. One primitive is required for each possible isotope of a given element.

## Bonds

Modification of the properties of existing bonds is straightforward. Some interpretation is required in order to interpret the meaning of the incoming selection, but all changes can be applied in a straightforward way to the molecule connection table. Connecting or disconnecting atoms which already exist can be done explicitly with a different primitive class.

### Set Bond Order

This class contains one primitive for each bond order supported by the molecular datastructure (e.g. 0, 1, 2, 3 and 4).

If the subject contains a single atom, this primitive is mapped to *New Bond with Order*, in the *Geometry *section, which creates a new atom and a new bond.

If the subject contains two atoms, and they are not currently bonded to each other, a new bond with the requested order is added between them.

Otherwise, all bonds between any two atoms within the subject set are set to the indicated bond order. Any of these bonds which previously had a specific stereo style is reset to the default non-stereochemical bond type.

### Set Stereo Style

This class contains one primitive for each explicit bond stereo-style supported by the molecular datastructure, which includes: inclined bonds (upward wedge); declined bonds (hashed wedge); and unknown stereochemistry (often drawn as a wavy line). When used correctly, these types are sufficient to unambiguously resolve most kinds of stereoisomerism.

Similarly to the *Set Bond Order *class, if the subject contains a single atom, this primitive is mapped to *New Bond with Stereo Style*, in the *Geometry *section.

If the subject contains two atoms which are not currently bonded to each other, a new bond of order 1 and the indicated stereo style is created. In the case of inclined or declined wedge bonds, the direction is arbitrary, and is defined by the current atom order.

Otherwise, all bonds between any two atoms within the subject set become the focus of the operation: the bond stereo style is set to the indicated type. If the indicated type is inclined or declined, then any of the affected bonds which are already of this type have their [from, to] order reversed, which inverts the meaning of the wedge, potentially altering the stereochemistry.

### Connect Atoms

Of all the subject atoms, any pairwise combination of two atoms which are not already bonded is considered. If there are any such atom pairs whose bond distances are approximately within the default bond distance (see Appendix 1) then all of these pairs are joined by adding a single bond between each pair.

If there are unbonded pairs, but none of them are close enough to the default bond distance, then only the closest pair of atoms is connected.

### Disconnect Atoms

Any bond for which both the participating atoms are a part of the subject is deleted. The atoms themselves are not otherwise modified.

## Deletion

Removal of atoms and bonds is straightforward, and requires only a small amount of logic to interpret the subject and apply the action to the molecule connection table.

### Delete Atoms

All of the atoms in the subject set are deleted, as are any bonds which are connected to them.

### Delete Bonds

Any bond which is between two atoms within the subject set is deleted. The atoms themselves are not modified.

### Delete All

All atoms and bonds are deleted.

### Merge Atoms

Each atom in the subject set is examined to see if it is particularly close to any other atom in the structure, typically set to a tolerance level which is significantly shorter than the default bond distance (see Appendix 1). For each of the subject atoms, a list is made of all other atoms to which the distance falls within the tolerance. From this list, one atom is selected to be retained, using the merging rules described in Appendix 2. The coordinates of the retained atom are set to the average position of the atoms in the list.

## Movement

While the unit primitives for grafting new fragments onto an existing molecular sketch are entirely sufficient for building up many complex molecules, there will always be structures which need to be fine tuned, or drawn with nonstandard parameters. This is often the case around heavily congested atoms for which there is no non-overlapping planar layout that adheres to common conventions.

Detailed control over individual atom positions is straightforward to implement, but care is needed to ensure that the primitives accomplish common tasks with a minimal number of invocations.

### Move Atoms

The subject atoms are moved in a specific direction. There are twelve primitives in this class: four directions (left, right, up, down) by three extents (small nudge, large nudge, move to furthest extent).

The small and large nudges offset the X or Y coordinates of the subject atoms in the given direction by an offset, such as 0.1 or 0.5 Å.

When moving to the furthest extent, the distance needed to move the subject atoms 1 Å beyond any of the other atoms in the molecule is calculated, and used as the offset.

### Scale Atoms

Two primitives are defined for this class: grow and shrink, which correspond to scaling factors of 1.25 and 0.8, respectively.

If the subject contains any selected atoms, then a central point is determined from the average positions of the selected atoms, unless there is also a current atom or bond, in which case its central position is used instead. Each of the subject atoms has its position recalculated by scaling its distance from the central point by the scaling factor associated with the primitive.

If there are no selected atoms, but there is a current bond, then this primitive is mapped to the corresponding *Scale Bond *primitive.

### Scale Bond

As for the *Scale Atoms *class, two primitives are defined: grow and shrink, which correspond to scaling factors of 1.25 and 0.8, respectively.

The subject must include two atoms which are bonded to each other. Each side of the bond is assigned a weighting of 0, 0.5 or 1.

If the bond is acyclic, then the atoms of the connected components on either side of the bond are counted. If one side forms a component with more atoms than the other, then the smaller side is assigned a weighting of 1 and the larger side a weighting of 0. If both sides have the same size, or the bond is cyclic, then both sides are assigned a weighting of 0.5.

The bond length is scaled according to the scaling factor assigned to the primitive, and the weights that are assigned to both sides, i.e. if a side has a weighting of 0 it does not move. For acyclic bonds, when moving one side of the bond, all other atoms associated with that side are moved as well. For cyclic bonds, only the two atoms that make up the bond are extended.

**Figure 1:**
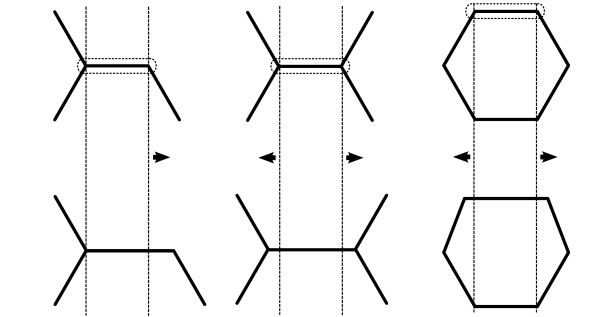
**Three examples of bond scaling**. The selected bond is outlined. The bonds are elongated in the directions indicated by the arrows.

Figure 1 illustrates increasing a bond length under three circumstances: unequal sides, equal sides, and a ring bond. The initial structures are shown on top, and the modified structures underneath.

### Flip Atoms

Two primitives are defined for this class: horizontal and vertical flip.

If the subject contains some number of selected atoms, then these atoms are flipped about the indicated axis. The origin of the axis is calculated as the average position of the selected atoms, unless there is also a current atom or bond, in which case its central position is used instead.

If the subject only contains a current atom or bond, then the whole connected component is used as the subject. If the subject is empty, then all atoms are used as the subject.

### Rotate Atoms

Primitives in this class are defined to be common rotation increments, such as ± 1°, ± 5°, ± 15° and ± 30°.

The position of the rotation centre is determined by the average position of the selected atoms, unless there is also a current atom or bond, in which case its central position is used instead. A further special case is defined: if there are no selected atoms, but there is a current bond, and one end of the bond is terminal, then the terminal atom is rotated about the position defined by the non-terminal end of the bond.

## Geometry

Because the information provided to the unit primitives cannot include spatial information such as bond direction, a crucial part of the design of the primitives is based on perception of atom geometry.

One of the most important sketching primitives is the ability to create a new atom which is bonded to an existing atom. With a traditional user interface this is done by using the mouse to drag a bond line in a particular direction, thus specifying bond angle and distance. Lacking such input, it is necessary for algorithms to be able to estimate the geometry of the atom, and from it, the most likely directions for a new bond.

**Figure 2:**
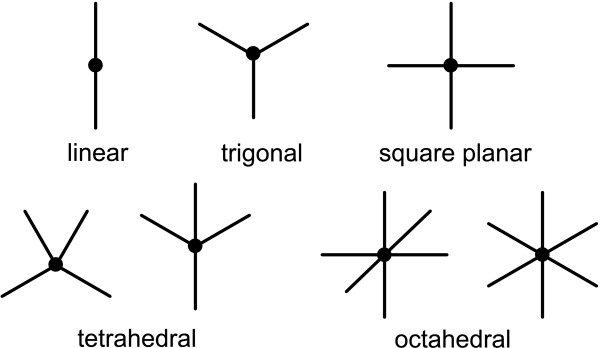
**Common geometries found in molecular sketches**.

Fortunately there are only a handful of geometry templates which are commonly observed in molecular diagrams, for atoms in environments which are not constrained by rings or heavy congestion. In this work, seven geometry templates are used. These are shown in Figure 2.

Most atom environments, when unconstrained, are drawn with complete or partial occupancy of one of these geometries. By examining the immediate bonding environment of an atom, it is often possible to make a reasonable estimate as to which of these geometry templates is most appropriate - and more importantly, which would be most appropriate in the context of adding an additional bond to a new atom. The process of assigning probable geometry based on topology and partial geometry is explained in Appendix 3.

### New Bond with Order

**Figure 3:**
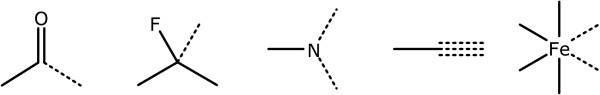
**Reasonable new bond geometries, based on existing layout and new bond order**.

When creating a new bond with a known bond order, the first step is to consider the subject atom with an additional bond with the requested order, connected to a newly created neutral carbon atom. In its new circumstances, the subject atom may have some number of preferred geometries. Consider the following cases shown in Figure 3: in each of these instances, the likely angles for the new bonds, indicated using dotted lines, are strongly suggested by the atom topology and the preexisting bond geometry.

**Figure 4:**
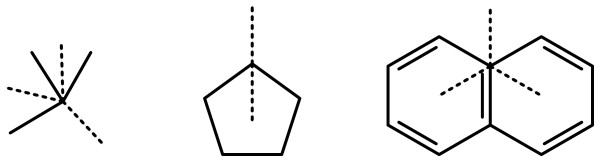
**Potential new bond geometries for atom centres which do not match a common template**.

In the examples shown in Figure 4, the geometry for the new bond is less clear, either because the incoming geometry is irregular or the valence is full. New bond positions are instead defined by the set of interior angles of adjacent neighbours.

Appendix 3 describes in detail the process of determining potential new bond angles. Once the list of angles has been generated, it is necessary to select one. For each of the angles, a point position is calculated by extending to the default bond length. The molecular congestion at each of these points is calculated (see Appendix 4), and the point which has the lowest congestion is used. A new carbon atom is created at this position, and a bond is created between the subject atom and the new atom.

### New Bond with Style

The new atom position is determined using the same method as for *New Bond with Order*, given that the bond order is 1. The newly created bond has the indicated stereochemical style, e.g. inclined, declined or unknown. For wedge bonds, the atom direction emanates *from *the subject atom.

### New Bond with Geometry

This class has one primitive for each of the 7 available geometries, which are shown in Figure 2. An attempt is made to create a new carbon atom and connect it to the subject atom via a single bond, using the indicated geometry.

The implementation is related to that of *New Bond with Order*, but more specific: if the current geometry about the subject atom does not match the indicated geometry with one angle missing, then the operation will instead be mapped to the corresponding *Set Geometry *primitive. If it does match, then all of the angles which are implied by matching the partial geometry are scored by calculating the congestion at the point of the implied new atom (see Appendix 4). The angle which corresponds to an atom with the least congestion is used to create the new atom and bond.

### Set Geometry

This class has one primitive for each of the 7 available geometries, as shown in Figure 2.

Each of the subject atoms is examined in the context of the requested geometry type. If the current geometry about the atom matches the requested geometry, with some number of missing bonds, then this primitive has no effect. If a partial match is not possible, the geometry about the atom will be refitted, if possible, in order to make it compliant with the requested geometry.

**Figure 5:**
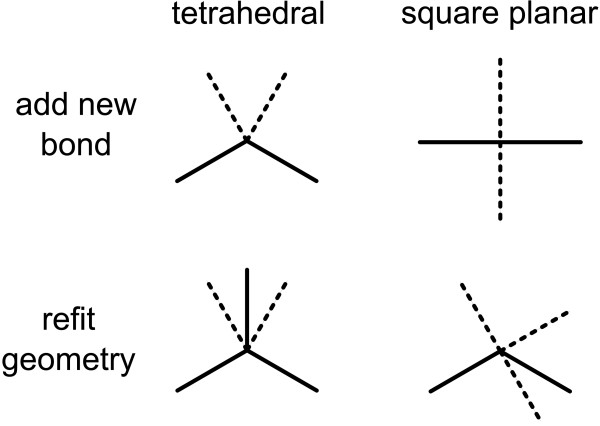
**Reconciling current geometry with a requested geometry**. Existing bonds are indicated by solid lines and proposed positions by dotted lines.

This primitive class is complementary with *New Bond with Geometry*. In Figure 5, for the top two examples, the existing bonds are able to be mapped in at least one way to the specified geometry, and so possible new bond angles are implied at the unoccupied positions. In the lower two examples, the geometries do not match, and the neighbours need to be refitted to the requested geometry.

For each pairwise combination of an existing bond and a bond from the geometry template, the template is rotated so that the two angles match. Of the remaining existing bonds, the angles are rotated so that they align with the closest angle from the rotated geometry template.

Cases which require moving of a bond which is part of a ring system are disallowed. If there are multiple ways to refit the geometry, that with the smallest overall angular displacement is chosen. If the geometry template is asymmetric, the process is repeated with its mirror image.

### Switch Geometry

One of the caveats of the methods by which the primitives select a new bond geometry is that there are often multiple choices which are quite similarly valid. Selecting the least congested position is the desired result more often than chance, but it is not uncommon for a more congested position to be preferred.

For this primitive, the subject must indicate a bond, and only one side of the bond must be terminal. The non-terminal end of the bond is examined, and its most likely bond geometry is estimated, as if the selected bond were not present (see Appendix 3). If no compatible geometry is found, or the only compatible geometry contains no available positions, this operation is not carried out.

**Figure 6:**
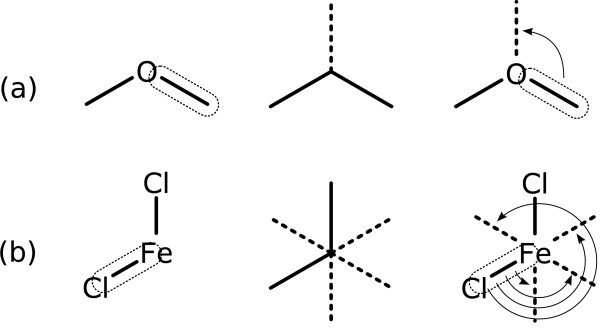
**Shifting a bond to a different position within the current geometry**. Existing bonds are indicated by solid lines and proposed positions by dotted lines. The current bond is outlined, and each of the possible rotations is indicated.

Any missing angles, which are non-degenerate and differ from the original bond angle, are considered to be viable new angles for the selected bond. Two examples are shown in Figure 6. In the first case, the bent ether fits the trigonal geometry, and a single distinct alternate position is available. In the second case, the metal centre fits the regular octahedral geometry, which presents 4 alternate positions to which the bond can be rotated. When there are multiple possibilities, the bond is rotated to the position with the smallest angular increment in the anti-clockwise direction, which ensures that repeated use of this primitive will rotate the bond through all of the available positions.

## Templates

The grafting of predefined template fragments onto an existing molecular structure is a vital part of the sketching process. Chemical structures include a number of diagram motifs which occur throughout the field, e.g. small rings of sizes 3 through 6 are all but ubiquitous, and rings which are neither square nor hexagonal are difficult to draw precisely. Besides rings and fused ring systems, there are a number of chains, branched chains, larger rings and functional groups which are particularly common. Almost all molecule structure drawing software has some number of predrawn templates, which can be added to the structure as a separate component, or attached to an existing component. Suggested default templates can be found in the Supplementary Information.

With a few exceptions, the algorithms needed for fusing an arbitrary structure with an arbitrary template fragment are non-trivial. Given the information allocated to the unit primitives described in this work, it is possible to specify information about the attachment site on the current structure, but not any information about which part of the template itself should be involved in the joining process.

Because practicing chemists often work on a series of projects for which particular structural motifs are frequently encountered, but not all of them are common throughout chemistry as a whole, it is also important to ensure that the list of available templates can be extended easily.

A standard convenience feature made available by almost all molecular drawing programs is the ability to copy portions of the current molecule onto a temporary container, often referred to as the clipboard, then paste them back later. For the purposes of this work, the clipboard should be considered as a single temporary template, i.e. the *copy *and *cut *actions that have become a standard part of the desktop metaphor place a single template onto the clipboard, and *paste *reads the template back out and applies it, using the same algorithm as is used for grafting predefined templates. The clipboard therefore shares the same primitive classes as the template functionality.

### Composing Templates

#### Create Template

This primitive class describes two operations: copying a molecular fragment to a temporary container, such as the system clipboard, and copying a molecular fragment to a persistent collection of fragments, such as a group of templates.

The template fragment itself is generated by considering the subject atoms to define a substructure, which is excised from the current structure.

If the subject atoms make up whole connected components, i.e. they are not bonded to any atoms which are not part of the subject, then the template fragment is taken to be the substructure in its entirety.

**Figure 7:**
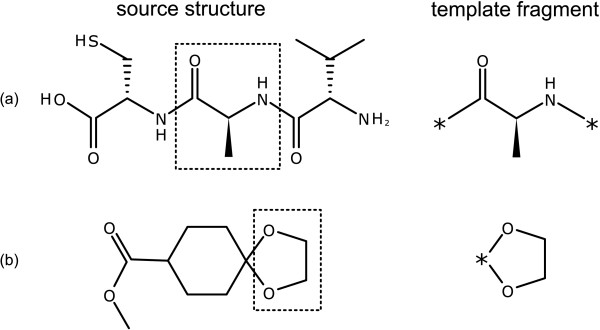
**Examples of excising template fragments**. The initial structure is shown on the left, with selected atoms outlined. The implied template is shown on the right, with guide atoms indicated by an asterisk.

Otherwise, all atoms which are immediately connected to one or more of the subject atoms are also included in the template fragment, but have their atom type converted into a placeholder atom label. In the examples shown in Figure 7, the placeholder atoms are denoted by the "*" symbol. These placeholder atoms are used as *guide atoms *for the primitive classes which make use of them, which is described below.

### Grafting Templates

A logical primitive is defined for each template that is available to the user, including the clipboard, if it contains a suitable molecular structure. The template fragment is an implicit parameter of the primitive. When the operation is instigated, several classes of behaviour will be investigated, depending on the nature of the template fragment and the subject.

The objective of the grafting procedure is to produce a list of putative new structures, each of which is a plausible way in which the template might be appended to the existing structure. Each of the following primitive classes is given an opportunity to generate some number of potential new structures, if appropriate.

As described previously, templates may have special *guide atoms*. The presence of these atoms introduces opportunities for special behaviour. Using guide atoms is a way to reduce the degrees of freedom when it comes to the possible types of outcomes, which is useful when a template was designed with the intention of using a specific attachment mode. It is also necessary when the attachment modes favour nonstandard bond distances or angles, which would not ordinarily be generated by the geometry perception algorithms.

#### Graft with No Connections

When there are no subject atoms, adding a template to the current structure is straightforward. It needs to be placed in an area where its atoms and bonds do not interfere with any that already exist, e.g. to the right and centre of the current structure.

All possible rotations of increments of 30° and 45° are included in the list of output structures. If the template contains guide atoms, they are stripped out.

#### Graft with Atom Connection

If there is one subject atom, this primitive applies. If the template contains guide atoms, they are stripped out.

New structures are generated using the following overall sequence:

1. Define *Mirror *to be the mirror image equivalent of the *Template*.

2. Loop over each atom, *N*, in the template fragment.

3. Direct Connect {*Template*, *N*}.

4. Direct Connect {*Mirror*, *N*}.

5. Bridge Connect {*Template*, N}.

6. Bridge Connect {*Mirror*, N}.

The connections are repeated with the mirror image of the template structure, in case it is not symmetrical. Generation of the mirror image is done by inverting one of the axes, e.g. *let x *= -*x*. If there are any bonds with *inclined *or *declined *stereochemistry, these are interchanged.

There are two main methods used for grafting templates using a single atom as the frame of reference. Direct connection involves overlaying the subject atom of the initial structure with the iterated atom (*N*) of the template fragment, and finding suitable angles by which to rotate the fragment. Bridge connection involves creating a new bond between the two atoms, rather than mapping them onto each other.

**Figure 8:**
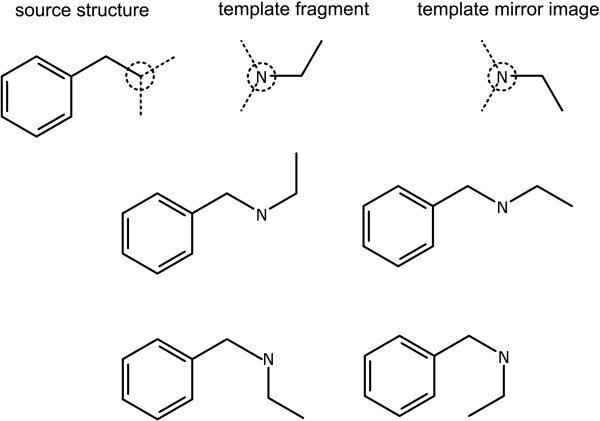
**Grafting two templates by direct atom-to-atom connection**. The input structures are shown with potential bond directions drawn as dotted lines. The unique set of grafted structures is shown underneath.

The direct connection algorithm starts by generating likely bond vectors for both sides, which is illustrated in the first row of Figure 8. The list of vectors on each side is composed from the same algorithms as used for determining the possible positions for a new single bond, as described for the *New Bond with Order *primitive class.

Both sets of angles are iterated over (θ_1 _and θ_2_). The template fragment is translated so that atom *N *is superimposed on top of the subject atom from the starting structure. The template fragment is rotated by θ_1_-θ_2_+180°, about the atom *N*. The two fragments are then combined, and the result recorded.

**Figure 9:**
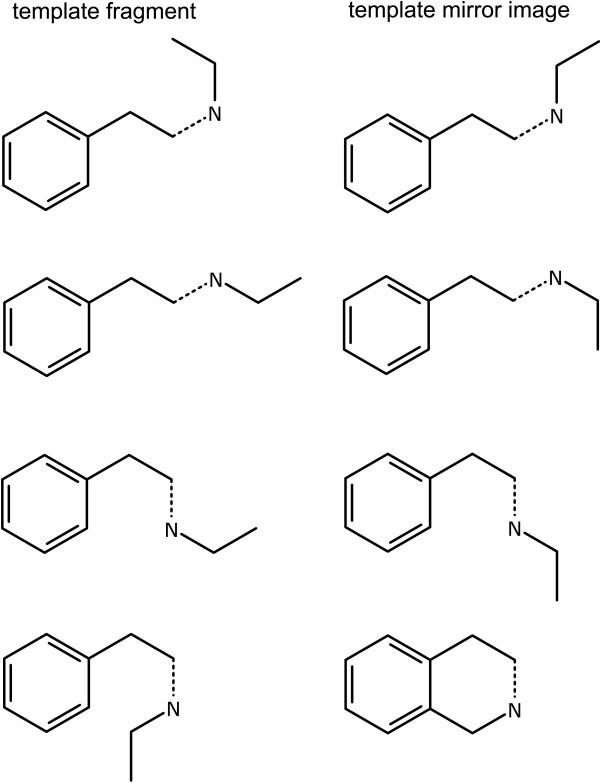
**Grafting two templates by bridged atom-to-atom connection**. The superimposed bonds are shown as dotted lines.

The bridge connection method involves essentially the same procedure, except that the template fragment is translated so that atom *N *is superimposed on top of a bond projecting from the subject atom with the angle θ_1 _and an extent equal to the default bond length. Rather than merging the two reference atoms together, a single bond is used to connect them. The results of this grafting are shown in Figure 9, which use the same input structure and template fragment as for Figure 8.

For both connection methods, and for all of the other primitives described in this section, the combination of the initial structure and a template structure can require some additional cleanup, since atoms and bonds can overlap. Overlapping atoms, and redundant bonds, are merged together, as described in Appendix 2.

#### Graft with Bond Connection

If there are two subject atoms, and they are bonded to each other, this primitive applies. If the template contains guide atoms, they are stripped out.

New structures are generated using the following overall sequence:

1. Define *Mirror *to be the mirror image equivalent of the *Template*.

2. Loop over each bond, *N*, in the template fragment.

3. Align Bonds Parallel {*Template*, *N*}.

4. Align Bonds Parallel {*Mirror*, *N*}.

5. Align Bonds Anti-Parallel {*Template*, *N*}.

6. Align Bonds Anti-Parallel {*Mirror*, *N*}.

**Figure 10:**
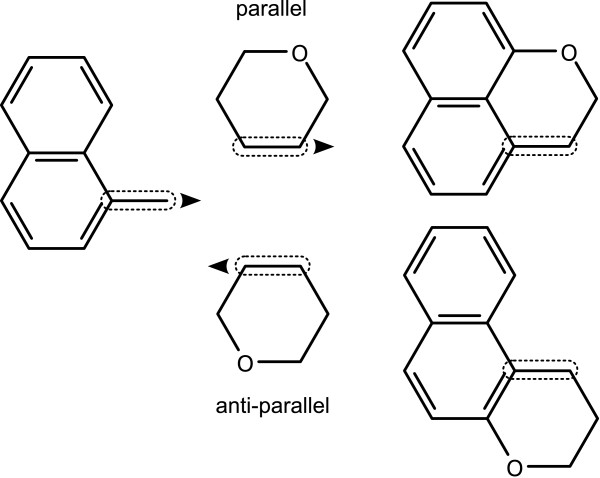
**Grafting two templates by aligning onto matched bonds**. The existing bonds that are superimposed are outlined.

Alignment steps are done by first translating the fragment so that the centroid of the bond, *N*, is superimposed on top of the centroid of the subject bond. The template fragment is then rotated so that the matched bonds are parallel or anti-parallel, which is illustrated in Figure 10.

In each case, the atoms associated with the matched bonds are merged together, using the method described in Appendix 2, except that the coordinates of the atoms from the input structure are always retained, which is relevant in cases where the bonds are of different lengths. Any remaining atoms which happen to overlap are merged as described in the appendix.

#### Graft with Multiple Connections

If there are more than two subject atoms, or there are two subject atoms and they are not bonded to one another, this primitive applies. If the template contains guide atoms, they are stripped out.

New structures are generated using the following overall sequence. The same steps are repeated with the mirror image of the template fragment.

1. Loop over each atom, *N_1_*, in the template fragment.

2. Translate the atom *N_1 _*onto the first subject atom.

3. Loop over each atom, *N_2_*, in the template fragment (*N_1 _*≠ *N_2_*).

4. Rotate the template fragment about *N_1 _*so that the direction of the *N_1_N_2 _*vector matches that of the first two subject atoms.

5. Match all remaining atoms.

**Figure 11:**
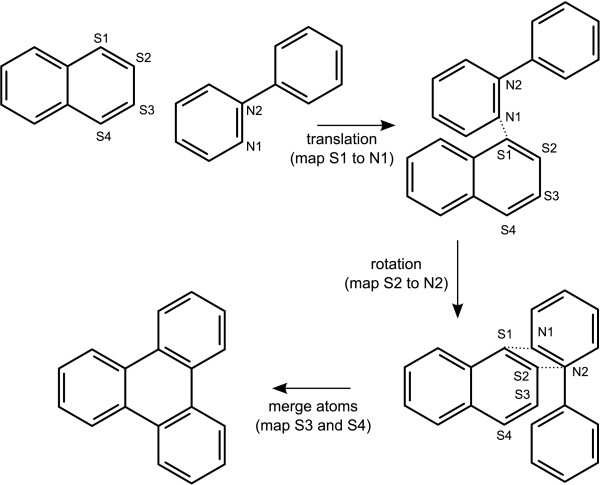
**Grafting two templates by matching multiple atoms**.

In step 4, the position of the first subject atom is used as the axis of rotation, so that the directions of the first two atoms are aligned. In the example shown in Figure 11, the subject consists of 4 selected atoms, which are labelled S1 through S4, while the two template fragment atoms of iteration are labelled N1 and N2.

If the position of the atom *N_2 _*from the template fragment does not now overlap the position of the second subject atom, then the graft is rejected. Once the first two atoms are aligned and matched, the remaining subject atoms are each required to overlap with one of the template atoms. If any of them do not, the graft is rejected.

Once all of the atoms are matched, the fragments are merged, as described in Appendix 2, and a new structure is added to the list.

#### Graft with One Guide Atom

If there is one subject atom, and the template contains one guide atom, and the guide atom has one adjacent neighbour, this primitive applies.

Grafting a template containing a single guide atom to a single subject atom has a similar effect to the *bridge connect *variant of the *Graft with Atom Connection *primitive, except with less degrees of freedom, because there is only one applicable template atom, and the projection direction and magnitude is defined by the guide atom.

**Figure 12:**
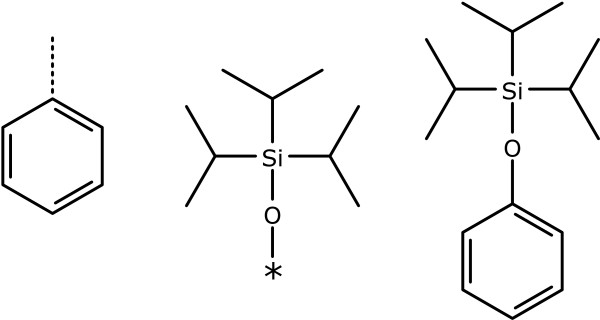
**Grafting of a template with a single guide atom**. The calculated new bond position for the substrate is indicated with a dotted line, and the guide atom of the template is indicated by an asterisk.

The list of projection angles emerging from the subject atom is calculated. These angles are matched against the angle formed from the guide to its neighbour. In the example shown in Figure 12, the input structure is benzene, with a single subject atom. The template is triisopropylsiloxy, where the geometry about the oxygen is drawn in a linear fashion, rather than the more commonly used bent orientation, due to congestion.

The bond distance is taken from the distance between the guide atom and its neighbour, rather than using the default bond distance. As with the other grafting primitives, the process is repeated with the mirror image of the template fragment. Once the grafting is complete, the bond connecting the guide atom to the rest of the template fragment is attached to the subject atom, and the guide atom is deleted. The remaining atoms are merged together if there is any overlap.

The main benefit of this primitive arises when a template is to be affixed using an irregular or non-obvious geometry, but it is also a way to ensure that a specific template connection point is used preferentially.

#### Graft with One Guide Bond

If there are two subject atoms, and they are bonded to each other, and the template contains one guide atom, and the guide atom has one adjacent neighbour, this primitive applies.

The template grafting is done by mapping the subject bond and the template fragment bond to each other. There are 4 base permutations, obtained by using the template fragment vs. its mirror image, and aligning the matched bonds in both parallel and anti-parallel fashion.

**Figure 13:**
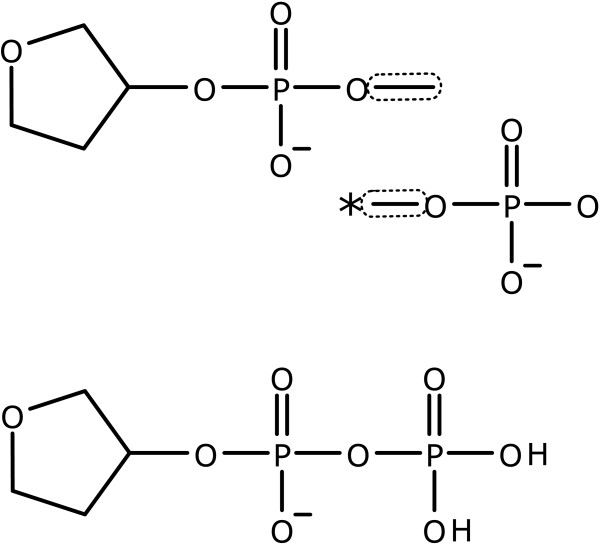
**Grafting of a template by matching a bond with a template atom connected to a single guide atom**. The bonds used to superimpose the structures are outlined. The grafted structure is shown underneath.

There is a further bifurcation if the two bonds differ in length: in one case the guide atom is mapped onto the first subject atom, and in the other case the atom adjacent to the guide atom is mapped onto the second subject atom, which is illustrated in Figure 13. On the left are the input structure (below) and the template fragment (above). On the right are the two distinct possible results, one for each of the two possible bond lengths. For each of the permutations, the mapping atoms and bonds are merged, as are any atoms which coincidentally overlap.

This primitive is useful as a constrained case of the *Graft with One Guide Atom *primitive, because both of the connection angles and the bond distance can be defined prior to the grafting process, which is particularly useful if the subject contains an irregular geometry or nondefault bond distance.

#### Graft with Multiple Guide Atoms

If there is at least one subject atom, and the template contains at least one guide atom, this primitive applies in the following cases:

1. There is one subject atom and one guide atom, and the guide atom has more than one neighbour.

2. There are 2 or more subject atoms, and the number of guide atoms is equal to the number of subject atoms.

**Figure 14:**
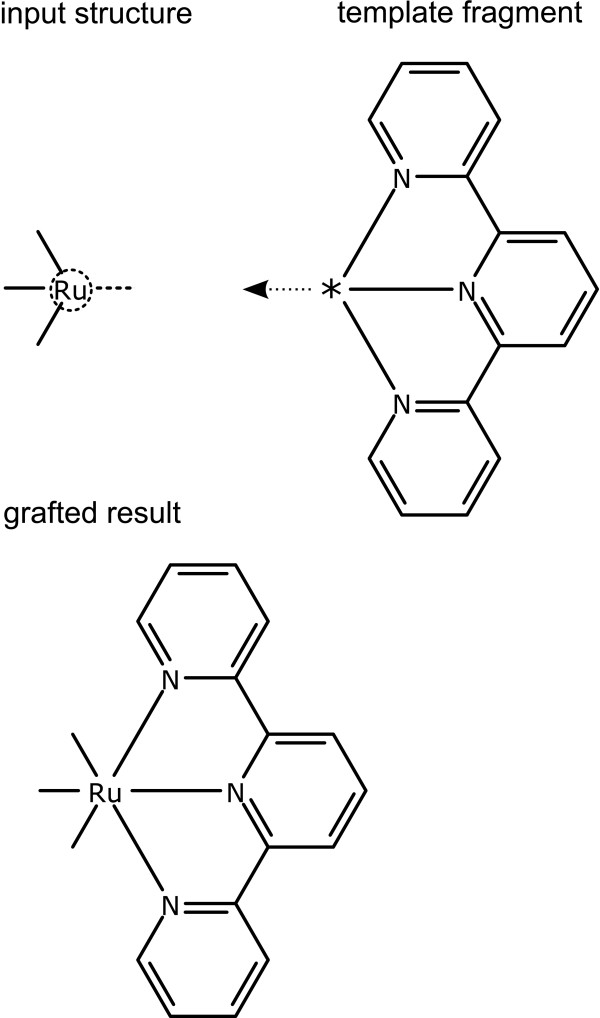
**Grafting of a template by matching an atom to a guide atom with multiple neighbours**. The input structure shows one of the possible bond directions as a dotted line. The template fragment is annotated by a dotted line showing the composite direction implied by the orientation of the guide atom.

The first case is dealt with in a similar way to the *Graft with One Guide Atom *primitive. Bond angle projections are generated from the source atom. For the template fragment, a median angle is generated, by considering the angles between the guide atom and the atoms adjacent to it. In the example shown in Figure 14, the subject is a ruthenium centre which has three substituents already, with a geometry that is compatible with a regularly drawn octahedral centre. One of the three possible projected bond angles emanating from the ruthenium atom is shown. The template is a tridentate ligand, in which the guide atom indicates the position of the chelated metal. The median angle between the guide atom and its neighbours is aligned anti-parallel to the substrate vector, to produce the result shown.

**Figure 15:**
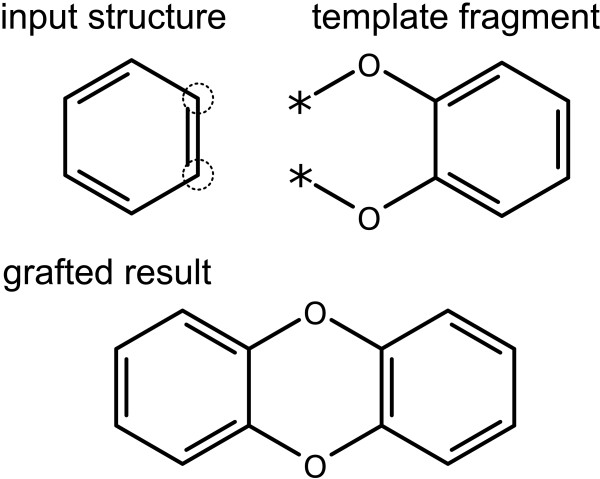
**Grafting of a template by matching multiple atoms to multiple guide atoms**. The selected atoms of the input structure are outlined, while the guide atoms are indicated using asterisks.

The second case is handled using the same technique as for *Graft with Multiple Connections*, except that the guide atoms are used to map the template fragment. As shown in Figure 15, the two guide atoms are aligned onto the two subject atoms.

When any successful matches are found, in which all of the guide atoms can be mapped onto a subject atom, the structures are merged together and the guide atoms are deleted.

### Applying Grafted Templates

After each of the primitives described above has had its chance to generate some number of putative new structures, the result list is processed. First, the list is trimmed such that whenever any two structures are found to be equivalent, one of the structures is removed from the list. The method used to decide whether two structures are equivalent is described in Appendix 5.

Once the unique set of structures is obtained, they must then be scored. The objective of scoring is to present the most feasible fused structures first, such that the user is most likely to see the desired result presented first and foremost. In cases where it is clear that some structures are significantly more reasonable than others (e.g. some of the results obey the Lewis octet rule, while the others feature pentavalent carbon atoms), then some of the results can be omitted.

The score for each grafted template result is computed by adding the following terms, where lower is better:

1. The total congestion of the molecule (see Appendix 4).

2. +1 for each atom that was merged with another atom due to positional overlap.

3. +50 for each atom of element type C, N, O, P or S which is clearly sp^3^, sp^2 ^or sp hybridised, and received a new bond which was not positioned at an angle 120° (sp^3 ^and sp^2^) or 180° (sp) from its neighbours, with a tolerance of 5°.

4. +1000 for every carbon or nitrogen atom with a total bond order of 5 or more.

5. -1 for every guide atom involved in the grafting process.

The scoring system heavily favours regular bond angles, prefers to add new atoms in less congested orientations, and encourages avoidance of certain types of impossible structures.

If the best available structure has a score of less than 1000, then all structures with a score of 1000 or more are excluded.

Once the scoring is complete, the structures are ordered so that the results with the lowest scores are shown first. For user interface purposes, it is appropriate to allow the user to traverse the list of potential structures, and select the desired result, if there is more than one. The best scoring result is frequently the intended result of the operation.

## Results

The unit primitives which have been described thus far provide a way to draw or modify structure diagrams with a small number of steps and a much lower input bandwidth than would be required from a conventional software package which relies on a pixel-perfect pointing device, such as a mouse or trackpad. The following examples illustrate the steps required in order to draw three molecules from scratch, using the primitives described in this work.

### Example 1: Aspirin

**Figure 16:**
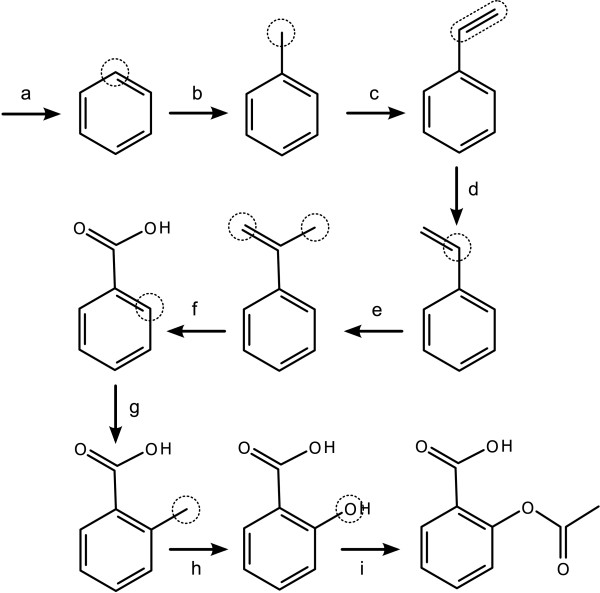
**Drawing aspirin by applying a stepwise sequence of primitives**. The selected or current atoms/bonds are shown as dotted outlines on the structure immediately preceding the primitive operation.

The first example, shown in Figure 16, illustrates the steps required to draw a simple organic molecule: acetyl salicylic acid (aspirin) [15]. Starting with an empty molecule, the 9 steps are shown below.

a. *Graft with No Connections*: benzene

b. *New Bond with Order*: 1

c. *New Bond with Order*: 2

d. *Switch Geometry*

e. *New Bond with Order*: 1

f. *Set Element Label*: O

g. *New Bond with Order*: 1

h. *Set Element Label*: O

i. *Graft with Atom Connection*: acetyl

### Example 2: Ingenol

**Figure 17:**
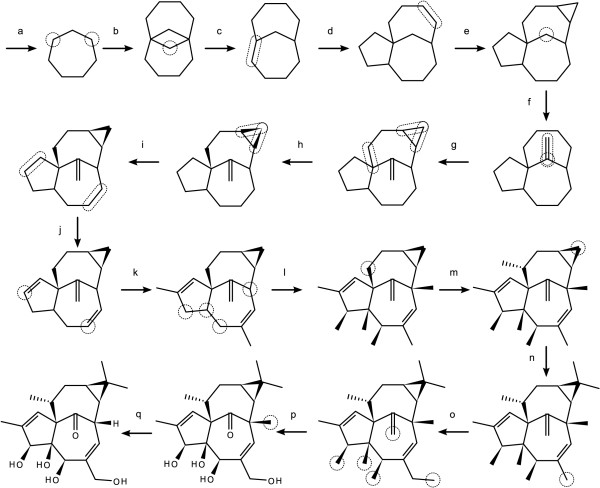
**Drawing ingenol by applying a stepwise sequence of primitives**. The selected or current atoms/bonds are shown as dotted outlines on the structure immediately preceding the primitive operation.

The natural product *ingenol *[16] can be drawn using the steps shown below, illustrated in Figure 17.

a. *Graft with No Connections*: cycloheptane

b. *Graft with Multiple Connections*: cycloheptane

c. *Delete Atoms*

d. *Graft with Bond Connection*: cyclopentane

e. *Graft with Bond Connection*: cyclopropane

f. *New Bond with Order*: 2

g. *Flip Atoms*: vertical. Note that the two atoms making up the double bond are *selected*, while the source of the terminal bond is the *current atom*.

h. *Set Stereo Style*: inclined

i. *Set Stereo Style*: inclined. Note that this reverses the direction of the already extant wedge.

j. *Set Bond Order*: 2

k. *New Bond with Order*: 1

l. *New Bond with Stereo Style*: inclined

m. *New Bond with Stereo Style*: declined

n. *New Bond with Geometry*: tetrahedral (variant #1). Note this primitive is issued twice, in order to create two new bonds.

o. *New Bond with Order*: 1

p. *Set Element Label*: O

q. *Set Element Label*: H

### Example 3: Organometallic catalytic intermediate

**Figure 18:**
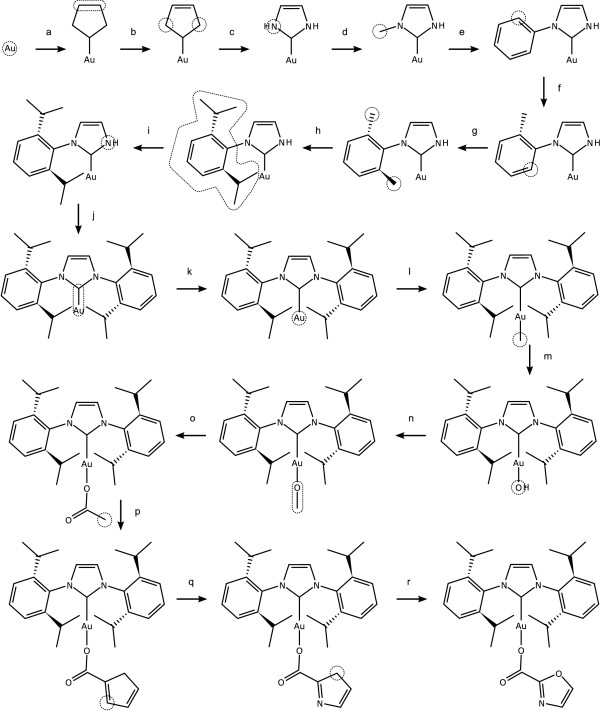
**Drawing an organometallic catalytic intermediate by applying a stepwise sequence of primitives**. The selected or current atoms/bonds are shown as dotted outlines on the structure immediately preceding the primitive operation.

The gold-based catalytic intermediate for a carboxylation reaction [17] is drawn using the steps listed below, starting from a single gold atom, and illustrated in Figure 18.

a. *Graft with Atom Connection*: cyclopropane

b. *Set Bond Order*: 2

c. *Set Element Label*: N

d. *New Bond with Order*: 1

e. *Graft with Atom Connection*: benzene

f. *New Bond with Stereo Style*: declined

g. *New Bond with Stereo Style*: inclined

h. *New Bond with Order*: 1. Note this primitive is issued twice, in order to create two new bonds for each of the selected atoms.

i. *Create Template*: copy to clipboard.

j. *Graft with One Guide Atom*: paste from clipboard.

k. *Scale Atoms*: grow

l. *New Bond with Geometry*: linear

m. *Set Element Label*: O

n. *New Bond with Geometry*: linear

o. *Graft with Bond Connection*: acetyl

p. *Graft with Atom Connection*: cyclopentadiene

q. *Set Element Label*: N

r. *Set Element Label*: O

## Conclusions

A collection of unit primitives for sketching molecular diagrams has been described. It is complete, such that complex molecules can be drawn by stringing together a series of these primitives. It is efficient, insofar as the more regular components of a chemical structure can be drawn using a small number of primitives, since only a very small amount of information must be transmitted from the user in order for the algorithms to infer the intent. Less regular features can be created by manipulating atom positions or angles using a variety of low level primitives, but a number of implicit shortcuts can be exploited to keep repetitive actions to a minimum.

Several representative examples have been shown, which illustrate the relatively small number of steps and low information content necessary to draw complex molecular geometries, and obtain publication-quality depiction layout coordinates.

The primitives described in this work provide the tools needed to build a user interface in which input is limited to selecting atoms and picking from menu choices. The interface can expose the primitives by several means, such as menu bars, icon toolbars, keyboard shortcuts, etc. As long as all of the primitives are conveniently available, the user interface will provide a complete and efficient molecule drawing tool.

These primitives have been implemented in a commercial software product called the Mobile Molecular DataSheet (MMDS), which is available for BlackBerry smartphones and iPhone/iPod/iPad devices [18]. Both versions offer access to the same basic primitives, but with different input techniques due to the differences between the devices.

While the motivation for this work arose from the need to provide molecular sketching capabilities on mobile devices with tiny screens and lack of accurate pointing devices, the set of primitives has applicability outside of this niche.

One analogous situation is found in web-based applications where the capabilities of the browser must be assumed to be a lowest common denominator, which requires that the web server do most of the work [19]. Under these circumstances, each user action can require a round trip to and from the server, which creates a low input bandwidth constraint, making the challenges quite similar to those faced by a mobile application. At the other end of the scale, sketcher applications being used on workstations or laptop computers with a mouse or trackpad could in some cases be improved by implementing a subset of these primitives, especially the template grafting algorithms, and providing them as additional tools.

The primitives described in this work were designed for the purpose of using them to compose a user interface, but they may find applicability as part of scripted processes. For example, when producing a series of chemical structures as part of a combinatorial library [20,21] or some other *de novo *series [22-24], it may be desirable to apply additional functionalisation, e.g. adding a new atom bonded to an existing atom, or joining two fragments to each other with some number of shared atoms. If the structural modifications were to be expressed in terms of these primitives, for a single substrate or an analogous series of them, the process of searching for a well arranged and aesthetically viable result could be significantly simplified.

## Appendices

### Appendix 1: Coordinates

Because a molecular sketch does not correspond to a physical model, the 2 D coordinates of the atoms are chosen so that they can be presented on a screen or a piece of paper, in such a way that chemists can quickly perceive the structural features and be able to ascertain stereochemistry from the layout and additional annotations.

The choice of units for the coordinates is arbitrary, but in this work, Angstroms are used. The default unit of distance between any two atoms is 1.5 Å, which applies regardless of atom and bond type.

Any two atoms that are closer than 0.2 Å are considered to be *overlapping*. Some of the algorithms described in this work consider overlapping atoms as a cue to merge two atoms together. Otherwise, the presence of overlapping atoms is generally considered to be an error state, since this makes visual perception of a diagram difficult.

### Appendix 2: Merging atoms and bonds

The template grafting algorithms described in this work mostly operate by joining specific atoms together. While these atoms are merged, bonded or deleted according to the algorithm, there is also the possibility for additional atoms to overlap. This situation is dealt with according to the following steps.

The composite structure is partitioned into its two sources, i.e. atoms that originated from the starting structure, and atoms that originated from the grafted fragment. Pairwise combinations of these atoms are examined to see if they overlap. When an overlap is found, one atom must be retained, and the other atom deleted. Before the other atom is deleted, its bonds must be reassigned to the retained atom.

The decision as to which atom to retain is made based on how *exotic *each atom is, which is calculated by assigning one point for each condition that applies:

- Having an element label other than carbon

- Non-zero charge

- Any unpaired electrons

- Non-default isotope abundance

- Specific non-automatic hydrogen count

The most exotic atom is retained, or the first one, if they are equally so.

During the merging process, it is possible to create duplicate bonds. A similar process is used to decide which bond to keep. The *exoticness *of a bond is assigned by one point for each of:

- Bond order other than 1

- Any stereochemical assignment

As for atoms, the first bond is retained in the event of a draw.

Other primitives besides template grafting also have need to merge together atoms which happen to overlap. In these cases the same procedure is used, but without the partitioning.

### Appendix 3

For many of the primitives described in this work, it is necessary to produce a list of potential angles for a new bond that will be created with a particular source atom and bond order in mind. When the list of angles produced contains the result which is desired by the user, the amount of effort required to draw the structure is considerably reduced.

The following sequence is used to obtain a list of potential new bond angles:

1. If the atom is isolated, return four angles, aligned on the X and Y axes.

2. Match the atom's bond topology to likely geometry templates (see Figure 2), and if any matches are possible, these are used.

3. Otherwise, return a series of median-cut angles.

Bond geometries are enumerated by considering the element, existing bond orders and the order of the new bond to be created. From this information, some number of plausible bond geometry templates may be produced, using the following sequence:

1. If the atom is terminal:

a. If the atom is either carbon or nitrogen, and the future bonding pattern is either alkyne-like or allene-like, classify as *linear*.

b. If the atom is not in the *s-*block or the *p*-block, classify as being either of the two *octahedral *geometries. These are versatile, as they form a superset of most of the other geometry templates.

c. If the atom is carbon, nitrogen or oxygen, classify as *trigonal*.

d. Otherwise, classify as either *trigonal *or *linear*.

2. If the atom is divalent, and the two current bonds are linear:

a. If the atom is in the *s*-block or the *p*-block, classify as *square planar*.

b. Otherwise, classify either of the two *octahedral *geometries.

3. Produce a list of geometries, depending on the element's position in the periodic table:

a. For non-element symbols (e.g. "E" or "X"), classify as either *trigonal *or *square planar*.

b. For *s*-block atoms, classify as either *trigonal*, *square planar *or either of the *octahedral *geometries.

c. If the atom is carbon, and the bonds are all single, classify as either *trigonal*, *square planar *or either of the *tetrahedral *geometries.

d. If the atom is carbon, and the bonds are not all single, classify as *trigonal*.

d. If the atom is in the *p*-block, and in the first 3 rows, classify as being *trigonal*, either of the *tetrahedral *geometries, or *square planar*.

f. If the atom is in the *p*-block, but not in the first 3 rows, classify as being any of the available geometries.

g. Otherwise, classify as either of the two *octahedral *geometries.

Once the possible classifications have been enumerated, each of them is checked to see if there is any way the current geometry can match it, within a tolerance of 2°. The first postulated geometry to achieve a match is given precedence. All of the nondegenerate vacant bond directions implied by the ways in which it can match the input structure are returned as the resulting list of possible new bond geometries.

If no matches were found, the list of current bond angles are sorted by bond angle. For each bond, the angle directly in between itself and the next angle in the list (or the first one, if already at the end) is calculated and added to the result list.

### Appendix 4: Congestion function

A number of the primitives use *congestion *to make decisions about where to place a new atom, bond or fragment, when an otherwise degenerate choice is available. The position which places new atoms as far away as possible from existing atoms is frequently preferable to the alternatives.

The congestion at a specific point is calculated by:

$$\sum_{i}\frac{1}{{\left(x_{i}-x\right)}^{2}+{\left(y_{i}-y\right)}^{2}+0.001}$$

where *i *iterates over each atom in the structure, and *x *and *y *denote the atomic position, where the subscripted variables are the positions of existing atoms.

The total congestion of a molecule is calculated by:

$$\sum_{i,j}^{i<j}\frac{1}{{\left(x_{i}-x_{j}\right)}^{2}+{\left(y_{i}-y_{j}\right)}^{2}+0.001}$$

where *i *and *j *iterate over all unique pairs of atoms.

### Appendix 5

Two structures A and B are considered equivalent or not according to the following algorithm:

1. If the number of atoms or bonds is different, the structures are different.

2. The structures are translated so that their centre positions are the same. Each atom in structure A is mapped uniquely to the closest atom in structure B, which must be within 0.2 Å.

3. If any atoms are not successfully mapped, the structures are different.

4. Every atom in structure A must be mapped to an atom in structure B which has the same element label, charge, unpaired electron count, etc. If any mapped pair of atoms are not the same, the structures are different.

5. For every bond in structure A, between atoms *a1 *and *a2*, there must be a corresponding bond in structure B, between atoms *b1 *and *b2*, where *a1*:*b1 *and *a2*:*b2 *are mapped to each other. If the bond stereochemistry type is not a wedge bond (inclined or declined), the inverse mapping, *a1*:*b2 *and *a2*:*b1*, is also permitted. If no such pair can be found, the structures are different. The matched bonds must have the same order and stereochemistry type. If not, the structures are different.

6. If no differences were found, the two structures are equivalent.

This comparison considers alternate tautomers and Kekulé resonance forms to be different species, which is desirable for sketching purposes. The determination of the equivalence of two sketches, as described above, should not be confused with methods used to determine whether two connection tables represent the same molecule, such as unique SMILES [25] or InChI [26].

## Supplementary information

A list of template fragments grouped into categories is provided [Additional file 1]. It is assumed that these templates are available as a minimum set, each as an individual primitive. These fragments are by no means comprehensive, but they provide a starting point for further customisation. The template fragments are also available as a collection of SD files [Additional file 2].

Additional examples of diagram drawing using the primitives described in this work can be found online [27].

## Competing interests

The author is the founder and president of Molecular Materials Informatics, Inc., which produces the Mobile Molecular DataSheet, a working implementation of the primitives described in this manuscript. This publication is a disclosure of the algorithms that form the basis of a commercial product.

## Supplementary Material

Additional file 1 **Suggested default template fragments**. A printable document containing diagrams of template fragments. Click here for file

Additional file 2 **Suggested default template fragments (SD files)**. A collection of MDL SD files, one per group, containing machine-readable data for each of the template fragments. Click here for file
