# Supplementary material for: Basic primitives for molecular diagram sketching
Source: J Cheminform. 2010 Oct 5;2:8. doi: 10.1186/1758-2946-2-8 (PMC2958898; doi:10.1186/1758-2946-2-8)
Supplement: Additional file 1 — Suggested default template fragments. A printable document containing diagrams of template fragments. [file 1758-2946-2-8-S1.PDF]

Template Groups

Small Rings

**Molecule**	**Name**
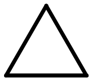	cyclopropane
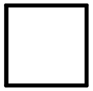	cyclobutane
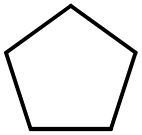	cyclopentane1
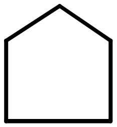	cyclopentane2
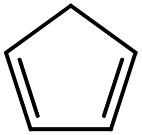	cyclopentadiene
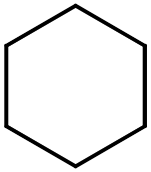	cyclohexane1
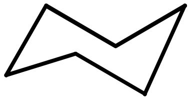	cyclohexane2
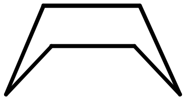	cyclohexane3
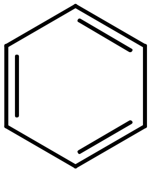	benzene
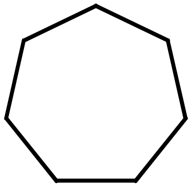	cycloheptane
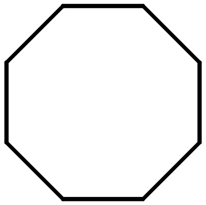	cyclooctane
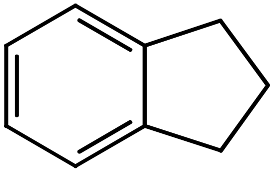	dihydroindene
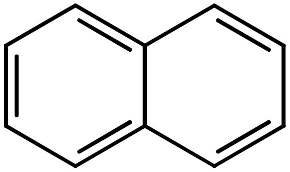	napthalene
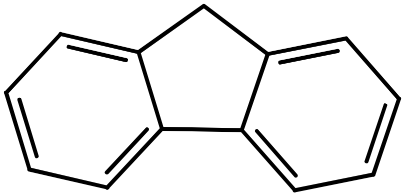	fluoroscene
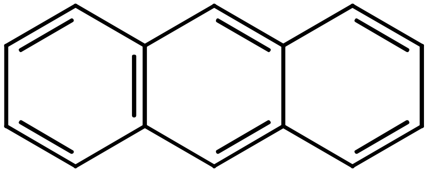	anthracene
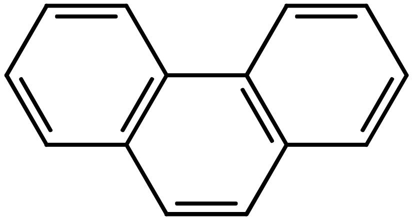	phenanthrene

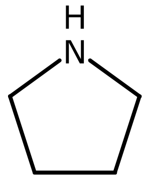

pyrrolidine

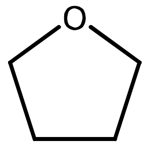

tetrahydrofuran

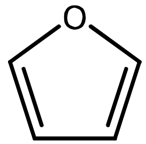

furan

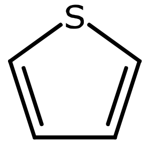

thiofuran

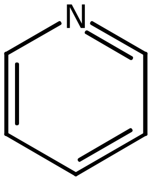

pyridine

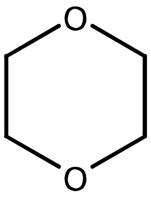

dioxane

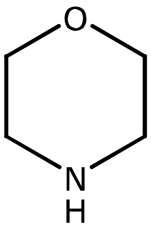

morpholine

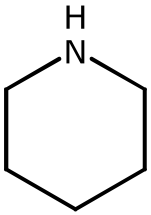

piperidine

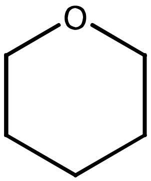

pyran

Terminal Groups

**Molecule**	**Name**
CH_4	methyl
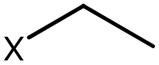	ethyl
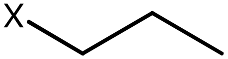	n-propyl
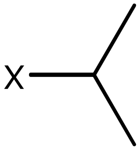	i-propyl
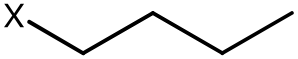	n-butyl
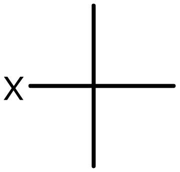	t-butyl
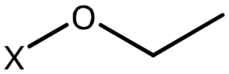	ethoxy
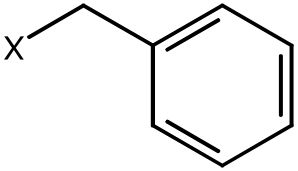	benzyl

Functional Groups

**Molecule**	**Name**
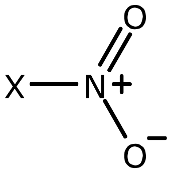	nitro
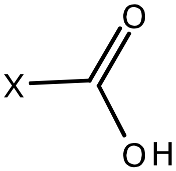	carboxyl
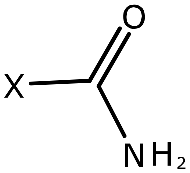	amide
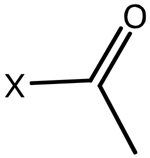	acetyl
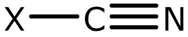	cyano
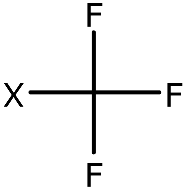	trifluoromethyl
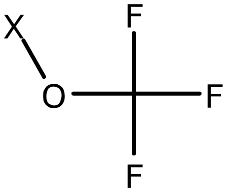	trifluoromethoxy

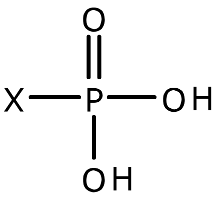

phosphoric

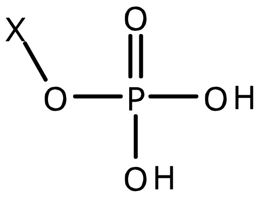

phosphoric

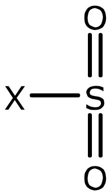

sulphonyl

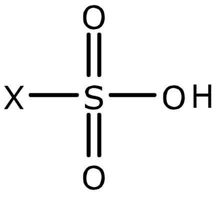

sulphonic

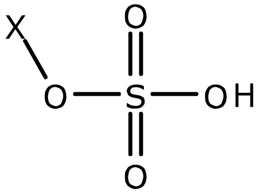

sulphonic

<u>Monodentate Ligands</u>

**Molecule**	**Name**
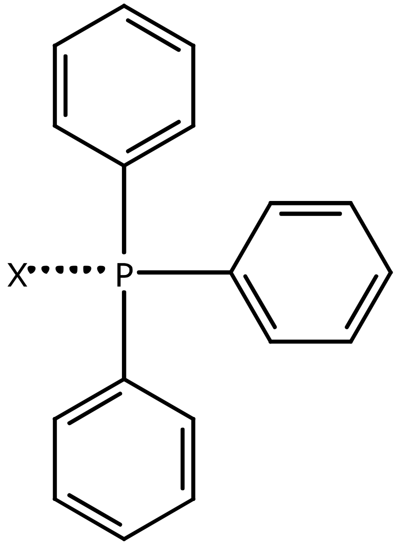	triphenylphosphine
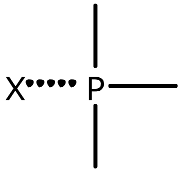	trimethylphosphine
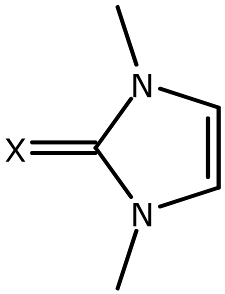	imidazolidine
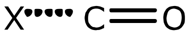	carbonyl

Bidentate Ligands

Molecule **Name**

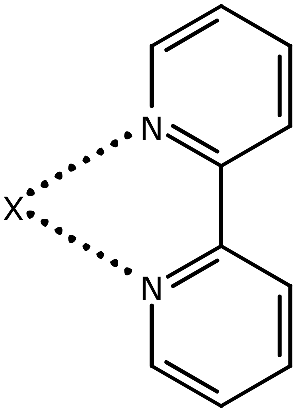

bipyridine

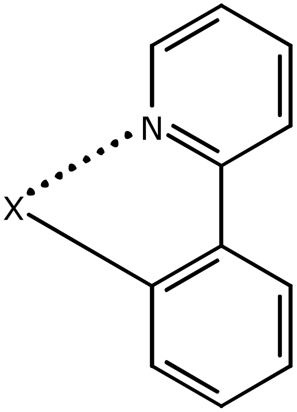

phenylpyridine

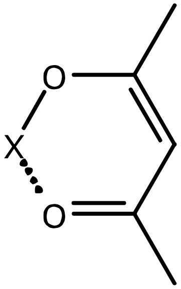

acetylacetonate

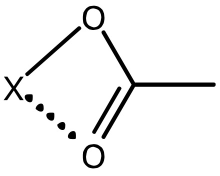

acetate

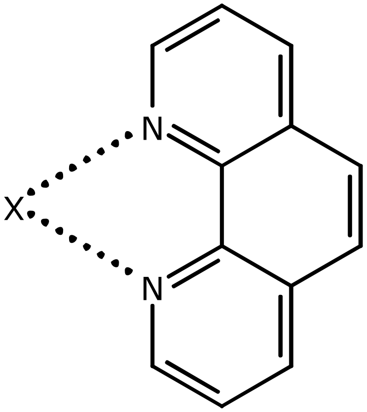

phenanthroline

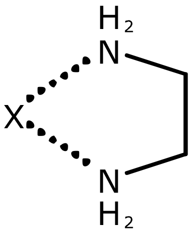

ethylenediamine

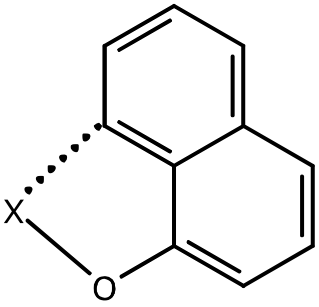

oxyquinoline

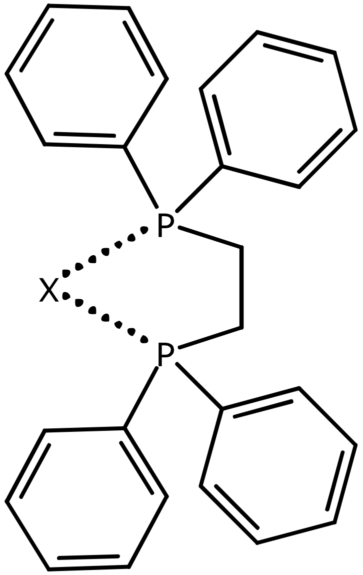

dppe

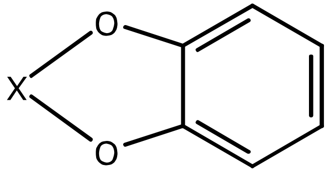

catecholate

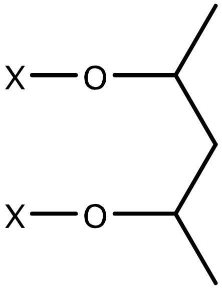

bridging acac

<u>Tridentate Ligands</u>

**Molecule**	**Name**
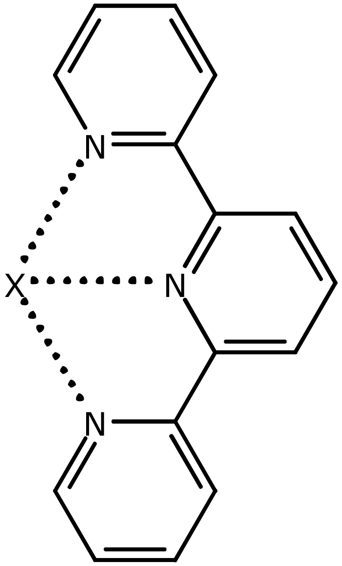	terpyridine
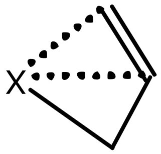	allyl
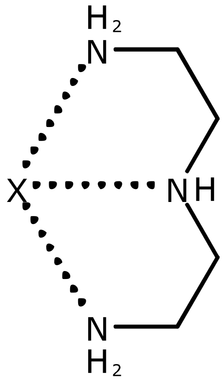	dien

Multidentate Ligands

**Molecule**	**Name**
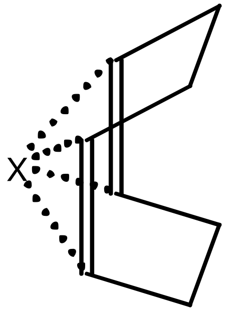	cyclooctadiene
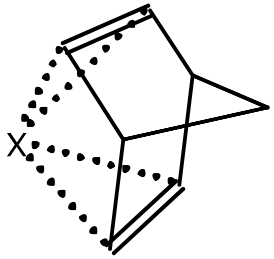	norbornadiene
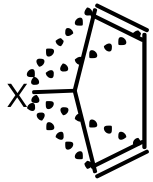	cyclopentadienyl
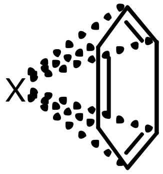	arene

Cage Complexes

**Molecule**	**Name**
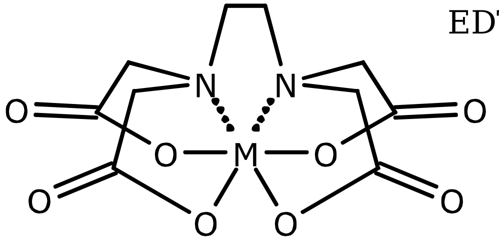	EDTA
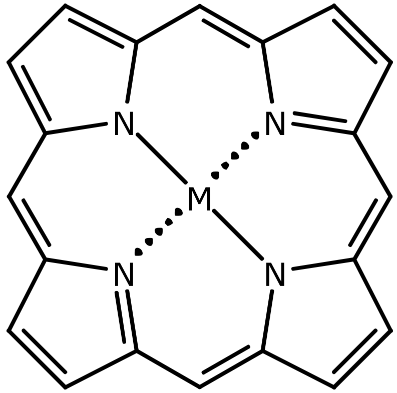	porphyrin
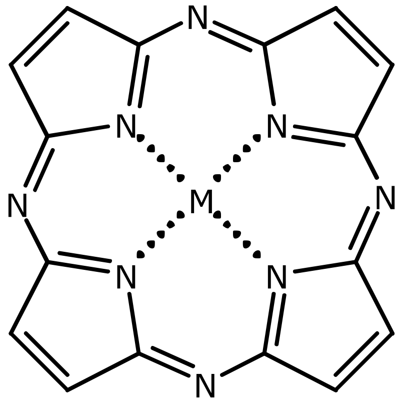	porphyrinoid
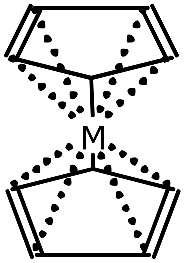	metallocene

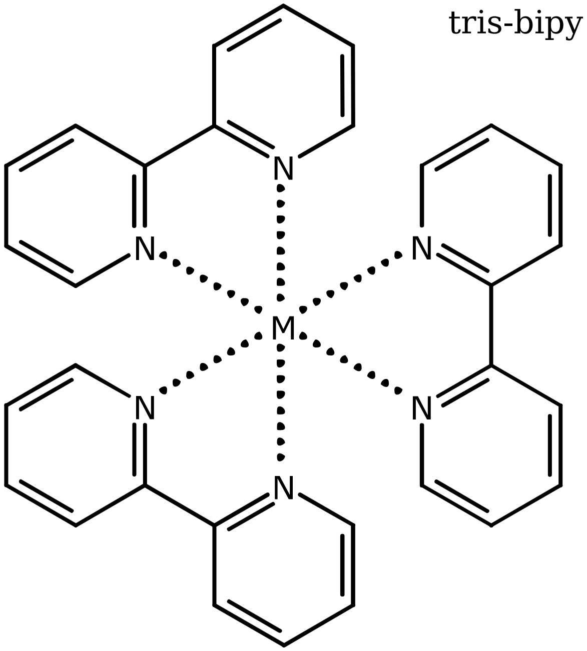

tris-bipy

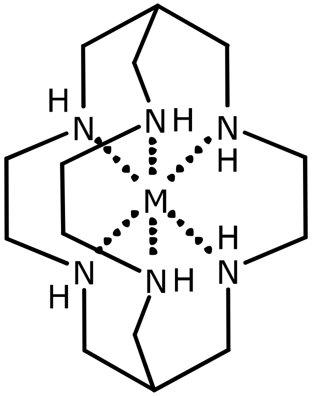

N6

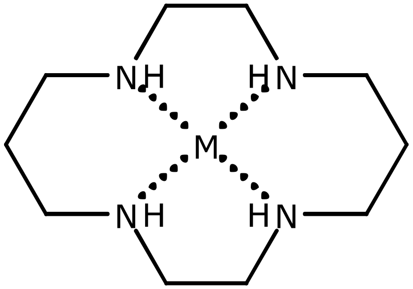

tren

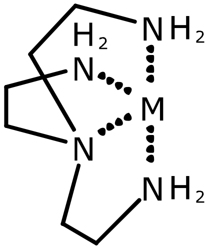

N4

Non-planar Rings

Molecule	Name
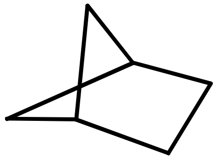	5-bridge-1
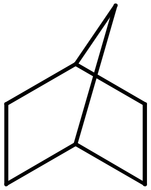	6-bridge-1
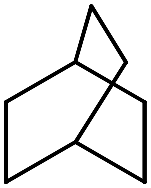	6-bridge-2
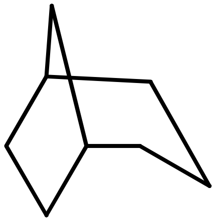	7-bridge-1
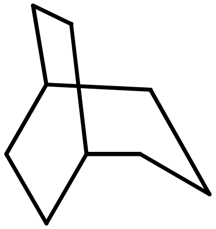	7-bridge-2
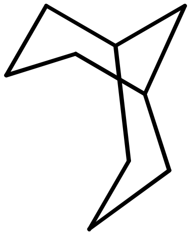	6-bridge-3
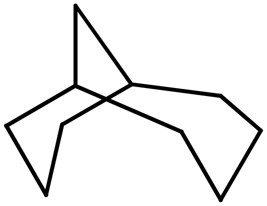	7-bridge-3
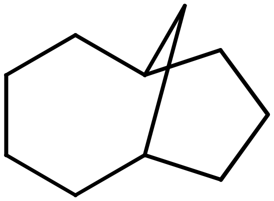	9-bridge-1
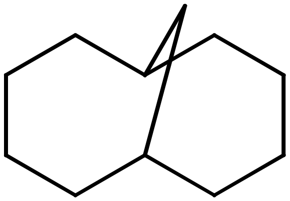	10-bridge-1
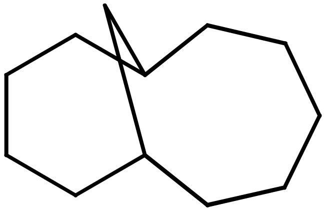	11-bridge-1
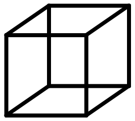	cubane
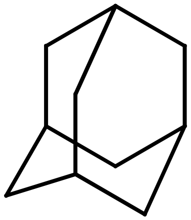	adamantane

Large Rings

**Molecule**	**Name**
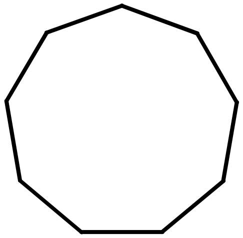	cyclo-9
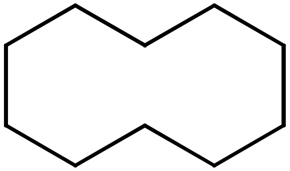	cyclo-10
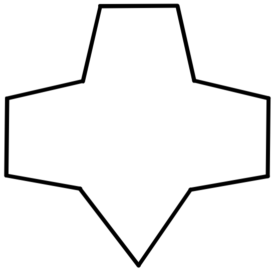	cyclo-11
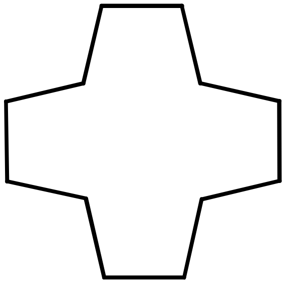	cyclo-12
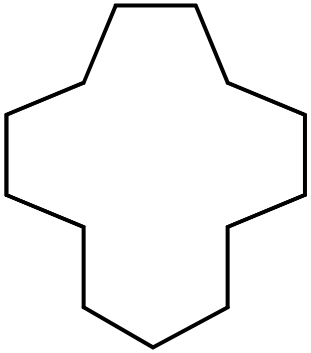	cyclo-13
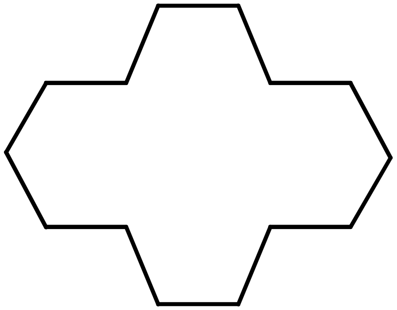	cyclo-14
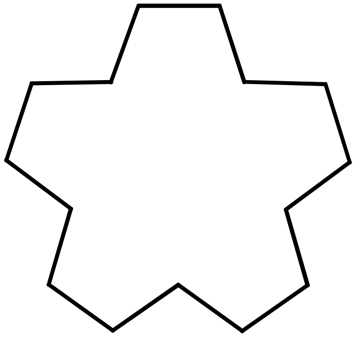	cyclo-15
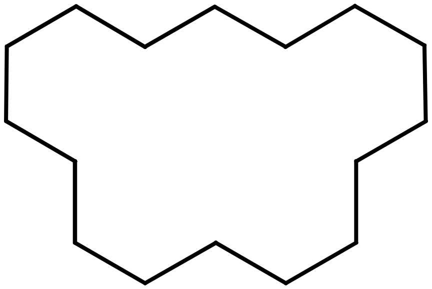	cyclo-16
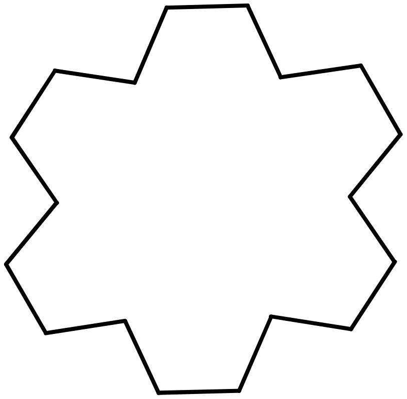	cyclo-18

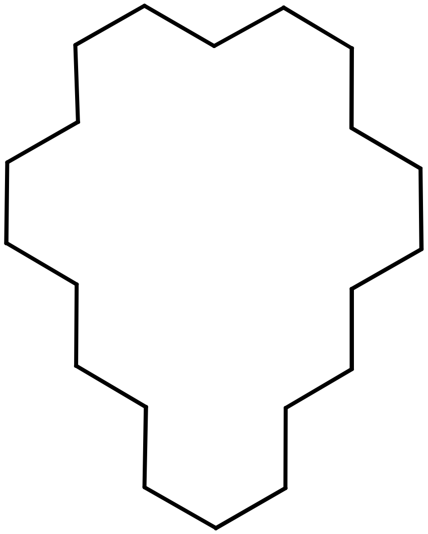

cyclo-20

Crown Ethers

**Molecule**	**Name**
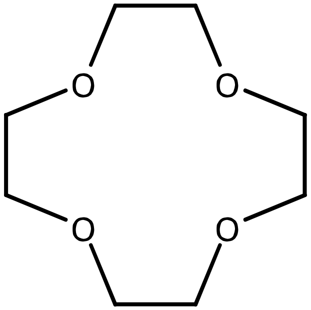	12-crown-4
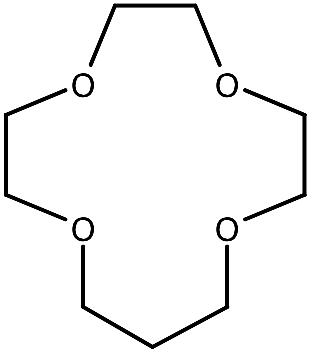	13-crown-4
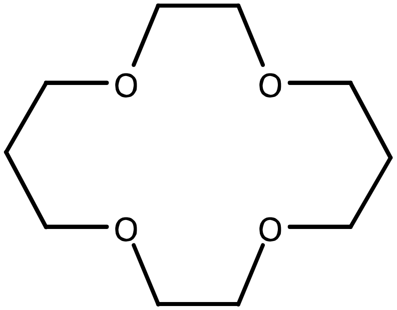	14-crown-4
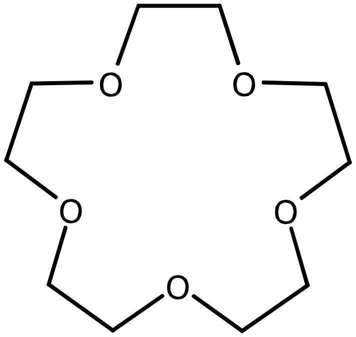	15-crown-5

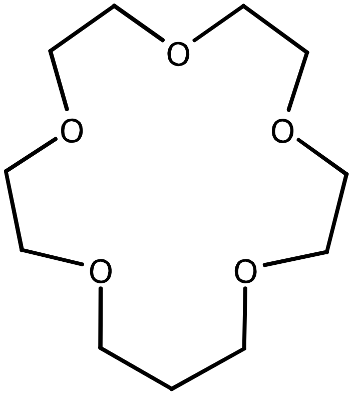

16-crown-5

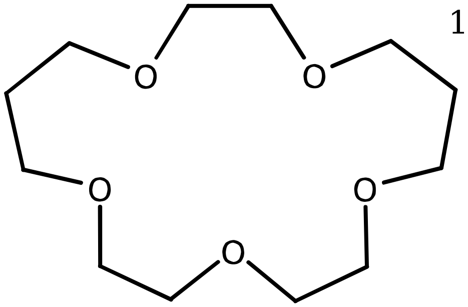

17-crown-5

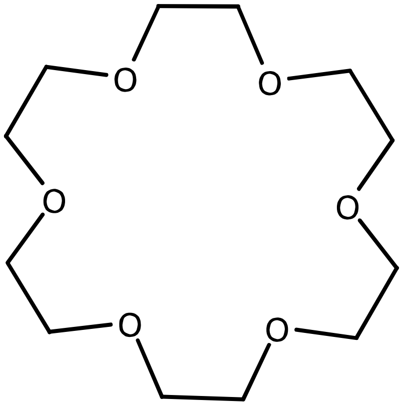

18-crown-6

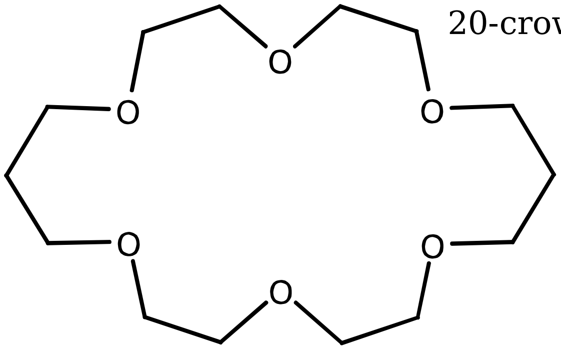

20-crown-6

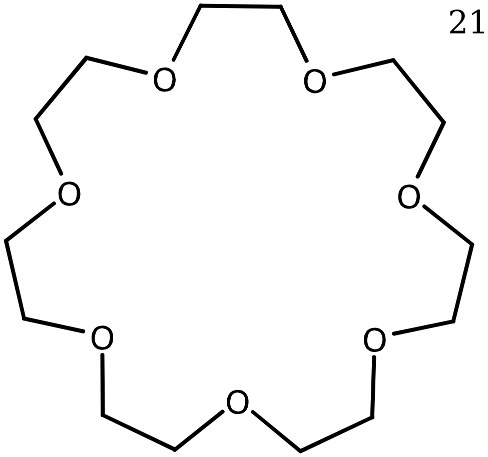

21-crown-7

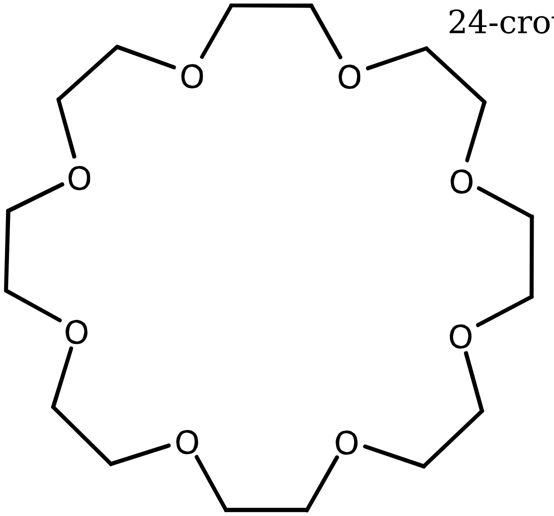

24-crown-8

Amino Acids

Molecule **Name**

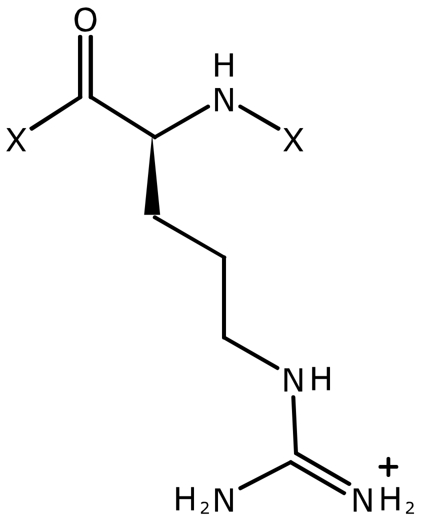

arginine

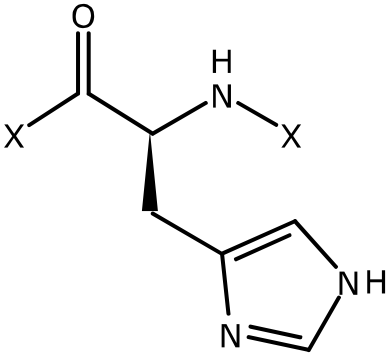

histidine

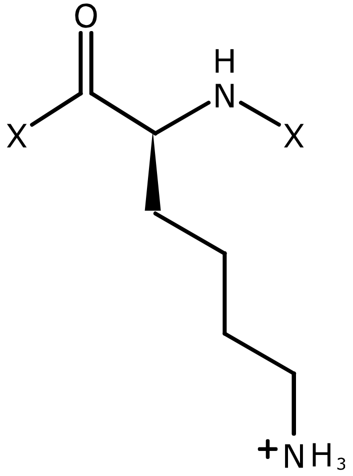

lysine

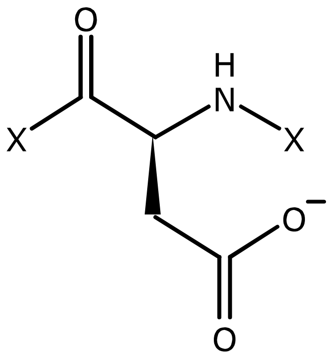

aspartic acid

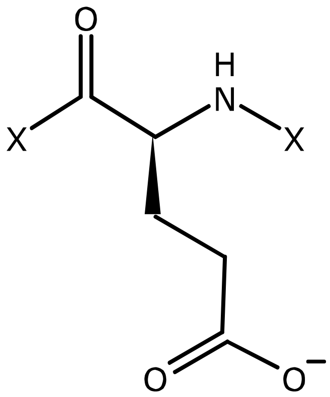

glutamic acid

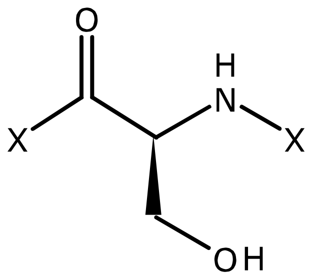

serine

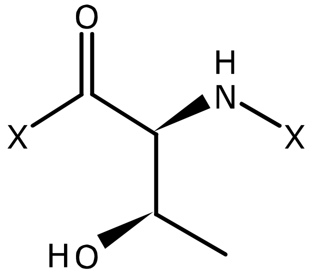

threonine

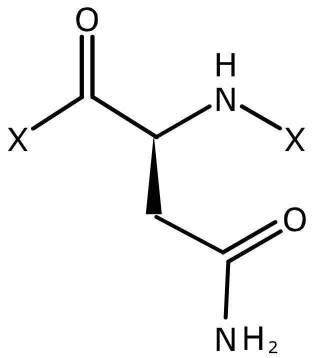

asparagine

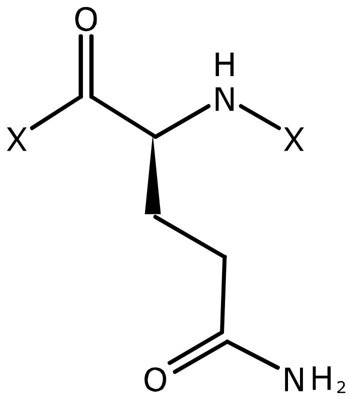

glutamine

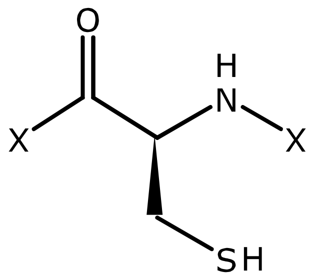

cysteine

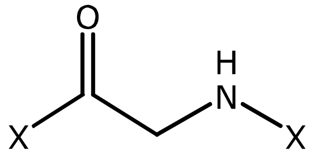

glycine

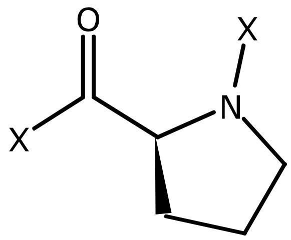

proline

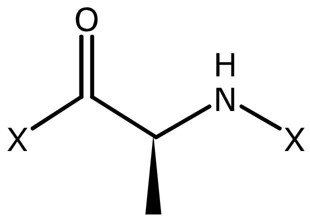

alanine

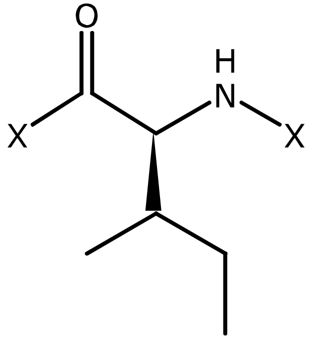

isoleucine

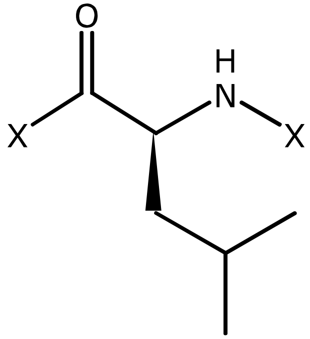

leucine

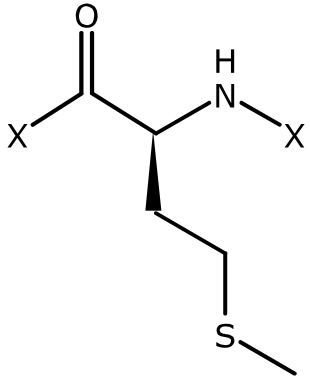

methionine

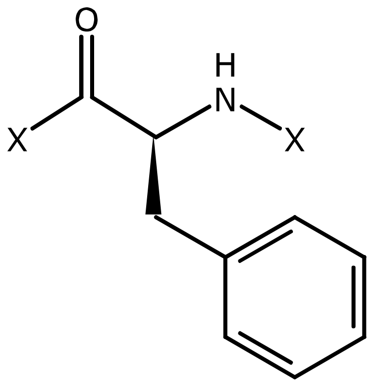

phenylalanine

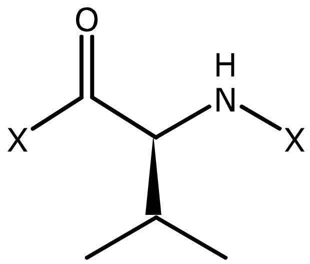

valine

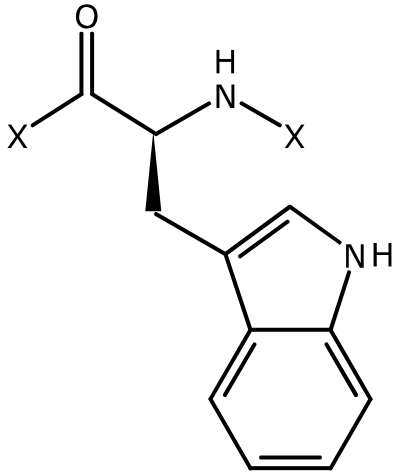

tryptophan

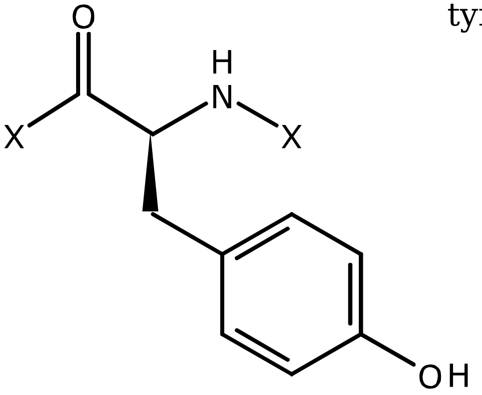

tyrosine

Biomolecules

**Molecule**	**Name**
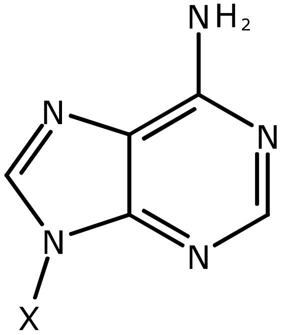	adenine
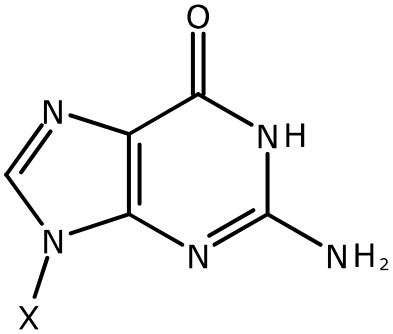	guanine
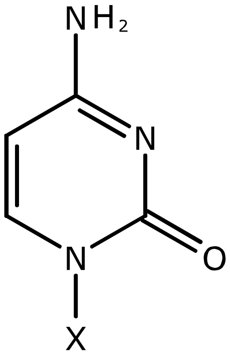	cytosine
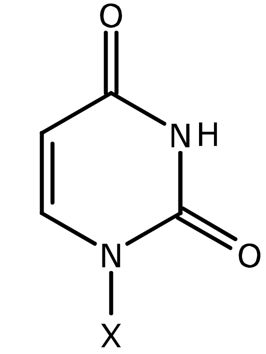	uracil
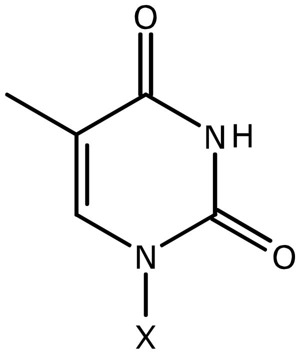	thymine
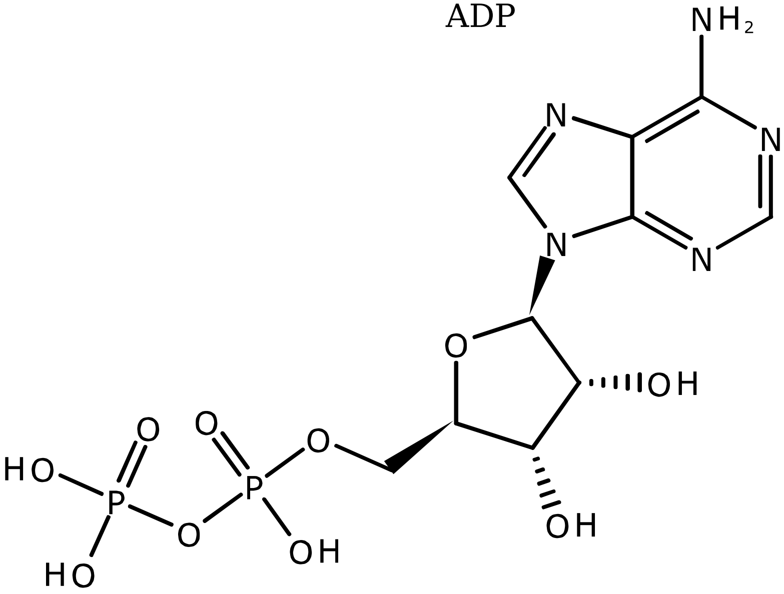	ADP

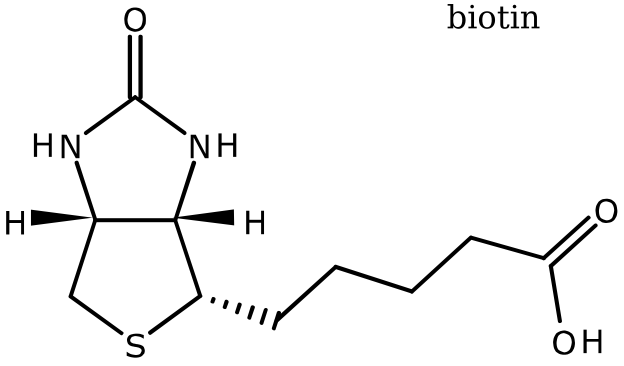

biotin

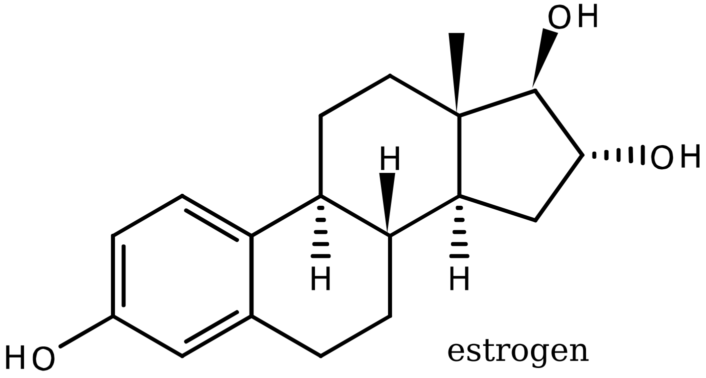

estrogen

<u>Saccharides</u>

**Molecule**	**Name**
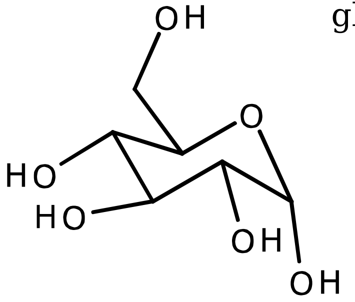	glucose alpha
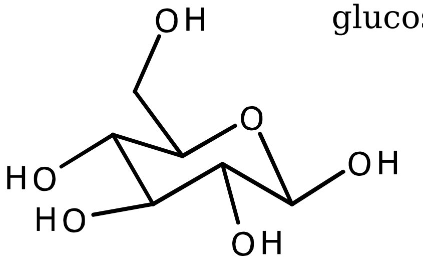	glucose beta
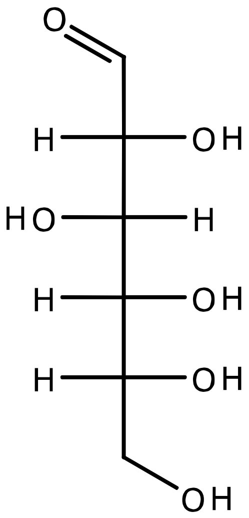	glucose chain

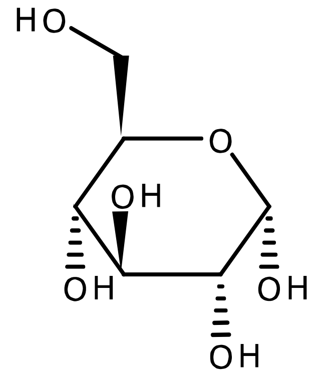

glucose alpha

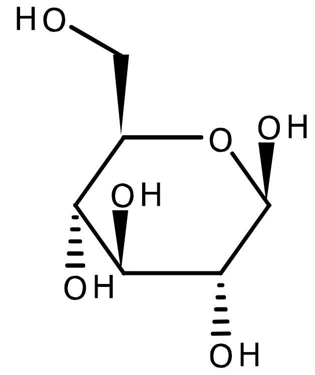

glucose beta

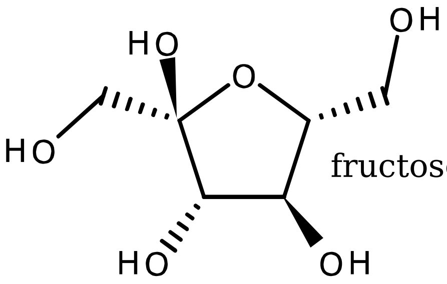

fructose alpha

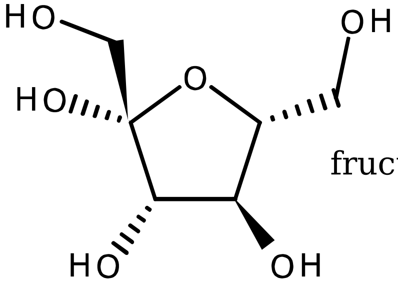

fructose beta
